# Truly form-factor–free industrially scalable system integration for electronic textile architectures with multifunctional fiber devices

**DOI:** 10.1126/sciadv.adf4049

**Published:** 2023-04-21

**Authors:** Sanghyo Lee, Hyung Woo Choi, Cátia Lopes Figueiredo, Dong-Wook Shin, Francesc Mañosa Moncunill, Kay Ullrich, Stefano Sinopoli, Petar Jovančić, Jiajie Yang, Hanleem Lee, Martin Eisenreich, Umberto Emanuele, Salvatore Nicotera, Angelo Santos, Rui Igreja, Alessio Marrani, Roberto Momentè, João Gomes, Sung-Min Jung, Soo Deok Han, Sang Yun Bang, Shijie Zhan, William Harden-Chaters, Yo-Han Suh, Xiang-Bing Fan, Tae Hoon Lee, Jeong-Wan Jo, Yoonwoo Kim, Antonino Costantino, Virginia Garcia Candel, Nelson Durães, Sebastian Meyer, Chul-Hong Kim, Marcel Lucassen, Ahmed Nejim, David Jiménez, Martijn Springer, Young-Woo Lee, Geon-Hyoung An, Youngjin Choi, Jung Inn Sohn, SeungNam Cha, Manish Chhowalla, Gehan A. J. Amaratunga, Luigi G. Occhipinti, Pedro Barquinha, Elvira Fortunato, Rodrigo Martins, Jong Min Kim

**Affiliations:** ^1^ Electrical Engineering Division, Department of Engineering, University of Cambridge, Cambridge, UK.; ^2^ Department of Engineering Science, University of Oxford, Oxford, UK.; ^3^ i3N/CENIMAT and CEMOP/UNINOVA, Department of Materials Science, NOVA School of Science and Technology, NOVA University Lisbon, Campus de Caparica, Caparica 2829-516, Portugal.; ^4^ Department of Materials Science and Engineering, Hanbat National University, Daejeon, South Korea.; ^5^ Advanced Material Research, Functional Textile Unit, EURECAT, Barcelona, Spain.; ^6^ Textile Research Institute Thuringia-Vogtland (TITV), Greiz, Germany.; ^7^ Bioelectronics and Advanced Genomic Engineering (BIOAGE), Lamezia Terme, Italy.; ^8^ Department of Chemistry, Myongji University, 116 Myongji Ro, Yongin, Gyeonggi-do 17058, South Korea.; ^9^ Solvay Specialty Polymers, Bollate, Italy.; ^10^ SAATI S.p.A, Appiano Gentile, Italy.; ^11^ Centre for Nanotechnology and Smart Materials (CeNTI), Vila Nova de Famalicão, Portugal.; ^12^ School of Materials Science and Engineering, Kyungpook National University, Daegu 41566, South Korea.; ^13^ Global Open Innovation Department, LG Display Co. Ltd., Seoul, South Korea.; ^14^ Lighting Applications, Signify, Eindhoven, Netherlands.; ^15^ Silvaco Europe, St. Ives, UK.; ^16^ Relats S. A., Barcelona, Spain.; ^17^ Henkel AG & Co. KGaA, Düsseldorf, Germany.; ^18^ Department of Energy Systems, Soonchunhyang University, Asan, South Korea.; ^19^ Department of Energy Engineering, Gyeongsang National University, Jinju, South Korea.; ^20^ Division of Physics and Semiconductor Science, Dongguk University, Seoul, South Korea.; ^21^ Department of Physics, Sungkyunkwan University, Suwon, South Korea.; ^22^ Department of Materials Science and Metallurgy, University of Cambridge, Cambridge, UK.

## Abstract

An integrated textile electronic system is reported here, enabling a truly free form factor system via textile manufacturing integration of fiber-based electronic components. Intelligent and smart systems require freedom of form factor, unrestricted design, and unlimited scale. Initial attempts to develop conductive fibers and textile electronics failed to achieve reliable integration and performance required for industrial-scale manufacturing of technical textiles by standard weaving technologies. Here, we present a textile electronic system with functional one-dimensional devices, including fiber photodetectors (as an input device), fiber supercapacitors (as an energy storage device), fiber field-effect transistors (as an electronic driving device), and fiber quantum dot light-emitting diodes (as an output device). As a proof of concept applicable to smart homes, a textile electronic system composed of multiple functional fiber components is demonstrated, enabling luminance modulation and letter indication depending on sunlight intensity.

## Teaser

Multifunctional textile electronic systems with functional fiber devices are demonstrated by fully automated integration.

## INTRODUCTION

A prosperous future of intelligent and smart environments could be revolutionized by a system architecture with unrestricted shape and unlimited scalability (*1*–*6*). Over the past few decades, flexible electronics technologies have been actively evolved to realize practical system applications such as display, energy harvesting/storage, and sensor networks (*7*–*10*). However, flexible multifunctional systems have yet to mature because of limitations in their mechanical stability under folding, bending, and rolling, depending on substrate types and stack materials/design, and limited scalability related to the dimension of fabrication equipment. Therefore, an alternative technology beyond flexible electronics requires the freedom of form factor, unrestricted design, and unlimited scale (*11*–*14*).

Textile platform with functional fibrous devices could offer an opportunity to realize previously unidentified types of electronic systems with a freedom of form factor (*1*, *3*, *15*), unlimited scalability (*2*, *16*, *17*), and high upgradability (*18*, *19*) after systemization. In this regard, fabrication technologies of fiber electronic devices and their integration strategies into textiles are needed (*20*–*22*). In particular, the development of automated processes including weaving and interconnection of fiber-based photonic, electronic, and energy devices into a single textile system for large-scale applications is at a nascent stage (*23*–*27*). In practice, there are considerable challenges for automated integration of fiber devices into textiles. The key challenges in these emerging fields are as follows: (i) fabrication processes of fiber-based devices, (ii) encapsulation of the devices, (iii) weaving of fiber devices into the textile system, (iv) interconnections between devices, and (v) design of the system architecture and its driving method for various functionalities. Many efforts have been made to overcome scientific and technical challenges in textile electronics through automated integration (*2*, *28*, *29*). However, an in-depth analysis and unified manufacturing process of functional fiber devices with nanomaterials, along with their integration in a single textile electronic system with multifunctional operation as information display, including photosensing input, emissive quantum dot (QD) lighting output, signal control between input and output, energy storage, and power management capabilities, have not been demonstrated yet.

Here, we demonstrate not only automated integration methods, including weaving and interconnection of fiber devices into textiles, but also machine-woven fully operational textile electronic systems with multiple types of fiber devices combined together in a single textile circuit and exhibiting a multitude of embedded signal control functions. Fiber devices including fiber photodetector (F-PD; as an input device), fiber supercapacitor (F-SC; as an energy storage device), fiber field-effect transistor (F-FET; as an electronic driving device), and fiber QD light-emitting diode (F-QLED; as an output device) are fabricated and encapsulated as basic elements required to build the textile system. The fiber devices are inserted into the textiles by automated weaving and interconnected via conductive threads by laser soldering. These processes, together with device architecture, are optimized to minimize damage to the functional devices. After integration, fiber devices are characterized to validate that our automated processes are applicable to large-scale textile electronic systems. Prototypes with the successive operation of F-PDs, F-SCs, F-FETs, and F-QLEDs are demonstrated for ambient assisted living (AAL) environments in smart homes.

## RESULTS

**Fig. 1.**
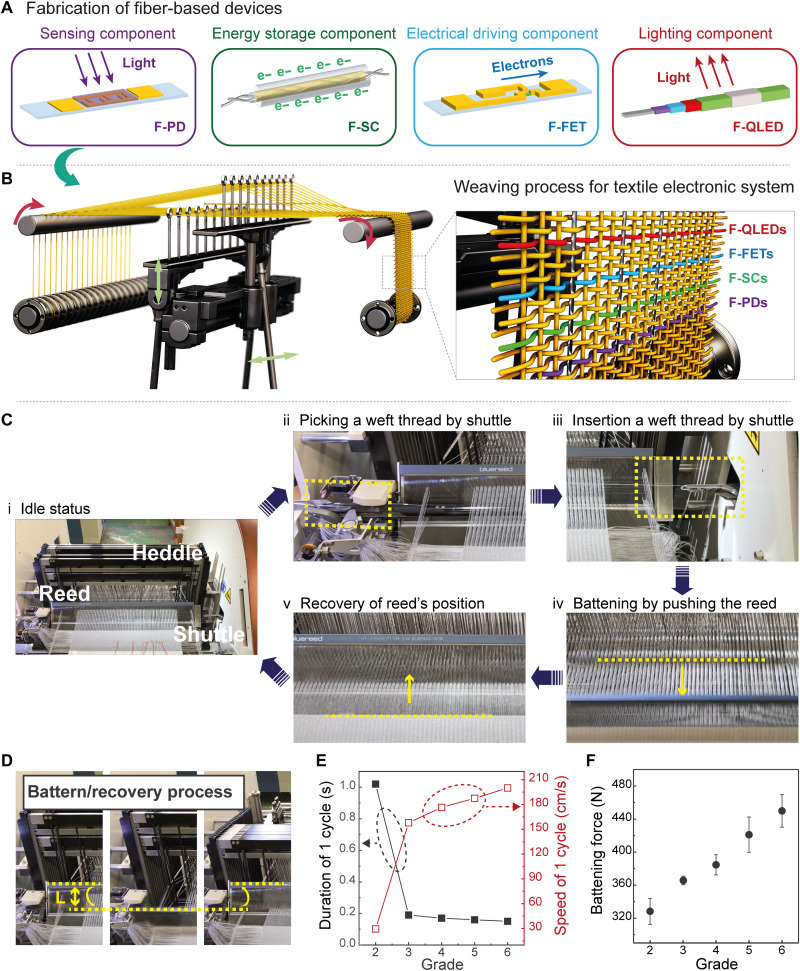
Automated weaving process for the textile electronic system. (**A**) Schematic illustration of the F-PD as a sensing component, F-SC as an energy storage component, F-FET as an electrical driving component, and F-QLED as a lighting component. (**B**) Schematic illustration of the programmable weaving process to integrate the fiber components into the textile. The fiber devices and conductive thread are inserted in the weft and the weft/warp directions, respectively, corresponding to the predesigned circuit diagram. (**C**) Photographs display the locations and movement of a shuttle and reed in the five steps during one cycle of weaving: idle status, picking a weft thread, insertion of a weft thread, battening by pushing the reed, and recovery of the reed’s position. (**D**) Photographs showing the change of the reed’s position for the battering/recovery process. The travel length of the reed in this system is 15 cm. (**E**) Duration of one cycle, speed of the reed, and (**F**) battening force at the various grades, which are speed units used in the program of the machine.

Figure 1 depicts an overview of the integration of fiber devices into the textile by automated weaving for the desired system architecture. We first developed the key fiber components and optimized their performance for integration into the electronics textile, as shown in Fig. 1A: (i) F-PD with on/off ratio of 10^4^ under ultraviolet (UV) irradiation (365 nm) (figs. S1 and S2); (ii) F-SC with a capacitance of 2.64 mF (figs. S3 and S4); (iii) F-FET with mobility of 12.8 cm^2^/V·s and on/off ratio of 10^7^ (fig. S5); and (iv) F-QLED with a luminance of over 900 cd/m^2^ (figs. S6 to S9). All these devices are fabricated by various techniques such as vacuum deposition or solution processing as a fiber form, which is defined by an aspect ratio of more than 30 with a width of a few millimeters. Then, the functional devices are encapsulated with protective layers to mitigate mechanical damage during the weaving process so that the performance of fiber devices is retained after integration (fig. S10).

### Integration of fiber devices into the textile by automated weaving and laser welding

The fabricated fiber devices are inserted in the weft (horizontal) direction together with conductive threads (55 ± 3 ohms/m; fig. S11) in the weft/warp (vertical) directions by automated weaving, corresponding to the design of the system architecture, as shown in Fig. 1B. The weaving process is composed of five steps: (i) idle status, (ii) picking a weft thread, (iii) insertion of a weft thread, (iv) battening by pushing the reed, and (v) recovery of the reed’s position (Fig. 1C, Materials and Methods, fig. S12, and movie S1). The reed travels distance (*L*), which is the length from the idle position to the battening position, in every weaving cycle (Fig. 1D). The mechanical force on functional fiber devices induced by the reed is controlled by the reed speed defined by the “grade,” which is the machine’s programmable speed unit (Fig. 1, E and F). The movement of the reed with a higher speed generates larger stress on the devices at the point of impact (setup of force measurement during weaving process in fig. S13). Combinatorial studies on the device characteristics and weaving conditions show that a mechanical force at the point of impact to the fiber devices higher than 365 N (reed speed higher than 150 cm/s) causes all devices to fail. Therefore, the weaving conditions were adapted to limit the impact force value at 330 N, which is generated by reed with a speed of 30 cm/s, as needed to minimize the mechanical stress on the fiber devices while still assuring high-throughput manufacturing.

**Fig. 2.**
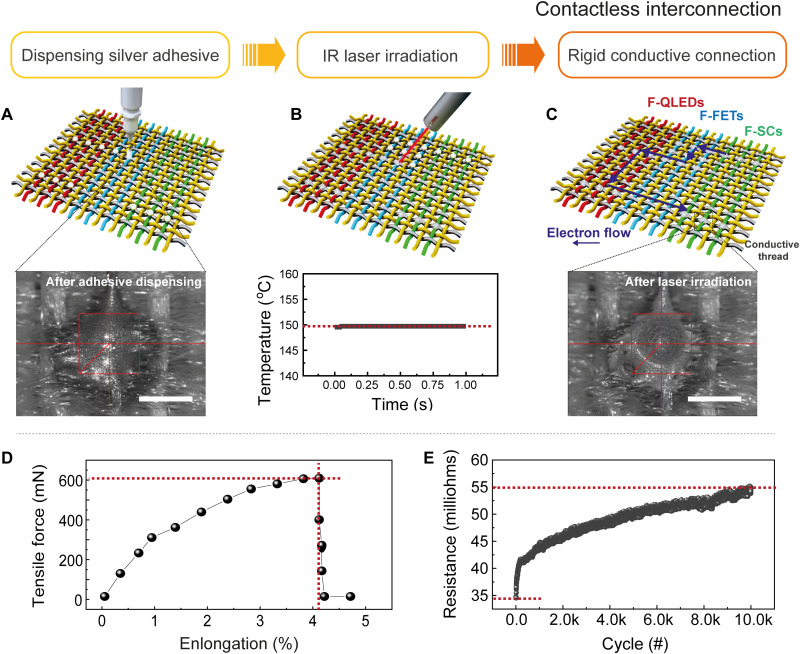
Automated interconnection process for the textile electronic system by laser welding. (**A**) Illustration of the automation system for dispensing conductive silver adhesive and optical image of the adhesive after dispensing on the targeted position. Scale bar, 1 mm. (**B**) Illustration of programmable laser illumination on dispensed adhesive (2.5 W, 1 s). The graph shows the temperature profile of the adhesive as a function of laser curing time. IR, infrared. (**C**) Illustration of the functional textile with electrical layout and an optical image of the adhesive after laser soldering. Scale bar, 1 mm. (**D**) Experimental evaluation of the mechanical properties of solidified conductive adhesive. (**E**) Electrical resistance of a series connection (thread–laser weld adhesive–thread) in the textile during abrasion test with respect to the number of abrasive cycling operation.

Interconnections between fiber devices and conductive threads in the textile are achieved by two-step contactless soldering using a 980-nm infrared laser (fig. S14). The first step entails dispensing a conductive silver adhesive onto the target position through a syringe (*D*_nozzle_ = 200 μm), as shown in Fig. 2A (movie S2). The second step is to cure it by laser (power of 2.5 W), and then, the silver adhesive is solidified at a temperature below 150°C within 1 s (Fig. 2B), which is found to be the optimal condition for avoiding thermal damage (fig. S15). Last, the fiber devices and conductive threads are interconnected with each other, corresponding to the desired system architecture (Fig. 2C). The mechanical stability is investigated by uniaxial stress of a solidified conductive adhesive that holds two conductive threads. The contact area of approximately 3 mm^2^ withstands a tensile force up to 600 mN (4% elongation; Fig. 2D). In addition, an abrasion test is conducted to confirm the durability of the electrical connection in the textile (setup for abrasion test in fig. S15). The electrical resistance is ~55 milliohms after 10,000 cycles (Fig. 2E). Note that the laser interconnection enables the demonstration of highly mechanically stable textile electronics systems.

### Characteristics of fiber devices after automated integration

**Fig. 3.**
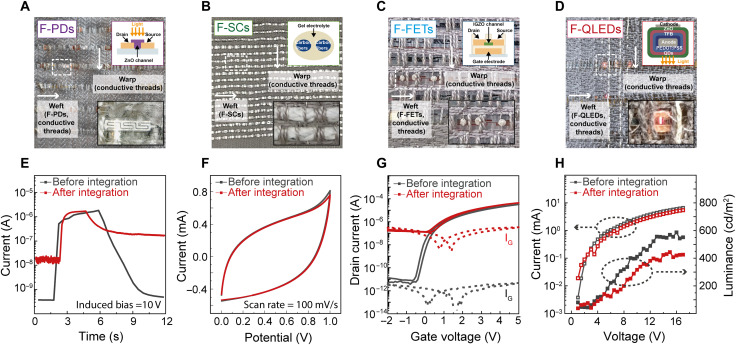
Evaluation of fiber devices after automated integration. Photographs of integrated (**A**) F-PDs, (**B**) F-SCs, (**C**) F-FETs, and (**D**) F-QLEDs into the textile in the weft direction. The inset in each photograph is a schematic illustration of the structure of the corresponding device. Characteristics of functional fiber components in the textile before and after automated integration. (**E**) Photocurrent of F-PDs at a *V*_DS_ of 10 V under the 365-nm UV irradiation with an intensity of 1 mW. (**F**) Cyclic voltammetry curves of F-SCs with a scan rate of 100 mV/s. (**G**) Transfer curves and gate current (I_G_) of F-FETs with *W*/*L* of 80/20 μm at a *V*_DS_ of 4 V. (**H**) Current and luminance of F-QLED as a function of the driving voltage.

We evaluate the performance of fiber devices after integration to verify the feasibility of both the weaving and interconnection approaches used. Photographs and schematic illustrations of fiber devices in the textile are shown in Fig. 3 (A to D). The functional textiles consist of cotton as a platform, conductive threads (weft/warp), and fiber devices (weft). Each electrode of the devices is interconnected to conductive threads inserted across the textile. Device characterization is carried out with electrical connections via conductive threads instead of directly contacting device electrodes. Photocurrent measurements of F-PD show that off-current increases to 10^−8^ A after integration, compared to 10^−10^ A before (Fig. 3E). Cyclic voltammetry curves indicate an unnoticeable capacitance change of F-SC (2.93 mF, 0.43% variation) after integration (Fig. 3F). The transfer curves of F-FET show that off-current increases from 10^−11^ to 10^−7^ A, whereas on-current is maintained (Fig. 3G). Given that the increase in off-current is accompanied by an increase in gate-leakage current of similar magnitude, the off-current increase can be attributed to degradation of the dielectric layer during the interconnection process (fig. S10). The current and luminance of a pixel of F-QLED are found to be approximately 75 % of their preweave values. Likewise, the luminance dims from 587 to 444 cd/m^2^ (Fig. 3H). To confirm the reliability of the fiber devices integrated into the textile through automated weaving and interconnection, tests for water resistance and long-term stability were conducted (fig. S17). The water resistance characteristic of fiber devices in textiles was studied under IPX7 conditions (1 m deep, 30 min) and revealed that all fiber devices exhibited unnoticeable performance change. The long-term stability of fiber devices was investigated by using the device in textile stored for 6 months in air and shows that all fiber devices maintain their initial characteristics without any noticeable degradations.

### Functional textiles fabricated by automated integration

**Fig. 4.**
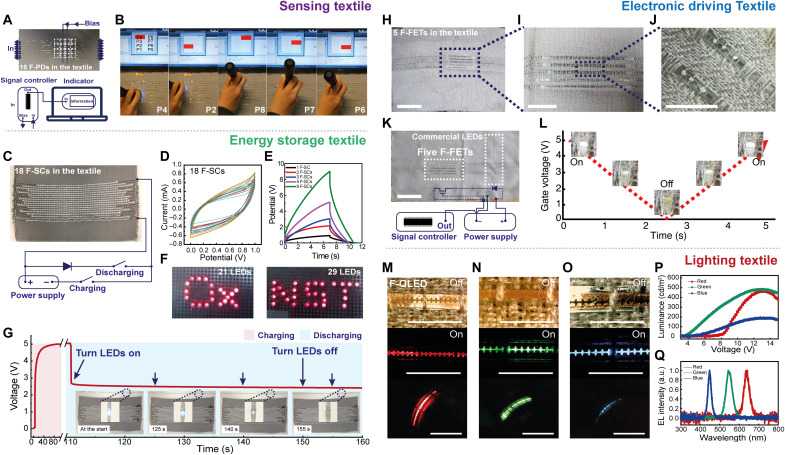
Photosensing, energy storage, electrical driving, and lighting textile fabricated by automated integration. (**A**) Sensing textile with F-PDs and a diagram of connections with a signal controller. (**B**) Photographs demonstrating UV light detection in multiple points of the photosensing textile. (**C**) Electrical configuration of energy storage textile. F-SCs are connected to provide 5 V of output voltage. (**D**) Cyclic voltammetry curves of 18 F-SCs to validate the energy storage performance after integration. (**E**) Charging and discharging properties of multiple F-SCs connected in series at a current of 0.7 mA. (**F**) Twenty-one and 29 commercial red LEDs, powered by energy storage textile with an output of 5 V. (**G**) Time-domain profile of output voltage for an array of F-SCs, which is connected to commercial white LEDs. (**H** and **I**) Integration of five F-FETs into the textile. Scale bars, 5 and 1 cm, respectively. (**J**) Highly magnified photograph showing two F-FETs after parallel interconnection of gate, source, and drain electrodes with conductive thread by laser soldering. Scale bar, 1 cm. (**K**) F-FETs in the textile are connected to a commercial LED, a signal controller, and a power supply. The signal controller is wired up to the gate electrode of F-FETs, controlling the brightness of the LED. Scale bar, 5 cm. (**L**) Profile of the gate voltage sweeping in the time domain for operating a white LED. The inset photographs show the brightness modulation of the LED as a function of gate voltage in the range from 0 to 5 V, at a *V*_DS_ of 5 V. Photographs show F-QLEDs woven in the textile for (**M**) red, (**N**) green, and (**O**) blue emission. Characteristics of (**P**) luminance and (**Q**) electroluminescence on red, green, and blue F-QLEDs. Scale bars, 1 cm. a.u., arbitrary units.

After demonstrating that each fiber device is able to withstand the integration processes on textiles, single-function textile systems were conceived, as presented in Fig. 4. Details of operation are demonstrated depending on fundamental applications, including (i) photosensing, (ii) energy storage, (iii) electronic driving, and (iv) light emission/character indication. A more detailed view of system level operation is shown in figs. S18 and S19 and movie S3.

The photosensing textile has eight UV detection positions (Fig. 4A). The generated signals from each photodetector are collected by the signal controller and then sent to an indicator (e.g., display or LEDs) for displaying information. An Arduino board is used as a signal controller. Once UV light (at 365 nm and with intensity of 1 mW) is irradiated on the photodetector, a current over 200 nA is generated through the corresponding F-PD, and the screen of a laptop displays the position where the UV light is detected (Fig. 4B). F-PDs are thus proven to be useful as a photosensing component for a textile system. The textile with 18 F-SCs is also demonstrated with a commercial LED and a charging/discharging circuit to confirm the feasibility of using F-SCs as an inbuilt power source (Fig. 4C). All F-SCs are measured in the range of 0 to 1 V at a scan rate of 100 mV/s. The capacitances of 18 F-SCs are evaluated, yielding a value of 2.64 ± 0.39 mF (Fig. 4D). Up to nine samples are connected, providing an output voltage up to 9 V (approximately 1 V per F-SC; Fig. 4E). It is verified that the energy storage textile can power 21 LEDs, 29 LEDs (Fig. 4F), and a strip of LEDs for 38 s (Fig. 4G), demonstrating the practical use of F-SCs for the textile electronics system.

The interconnection enables the electrical connection between the F-FETs and other electronic components, such as LEDs and a power supply with conductive thread by laser soldering (Fig. 4, H to K). As shown in Fig. 4L, the result demonstrates that F-FETs in the textile can control the brightness of a commercial LED, validating the functionality of electrical driving component for the textile system. Light-up textile is also demonstrated (Fig. 4, M to O). Red, green, and blue light emissions from F-QLEDs woven in the textile are observed with a peak luminance of 463, 482, and 188 cd/m^2^, and electroluminescence peak wavelength of 630, 540, and 450 nm, respectively (Fig. 4, P and Q). Single-function textiles that show qualified performance are crucial to realize multifunctional textiles by designing system architectures with automated system integration. (In addition, preliminary tests of textile system architectures for energy management have been conducted, as shown in fig. S20).

### Integrated textile electronic system for AAL applications

**Fig. 5.**
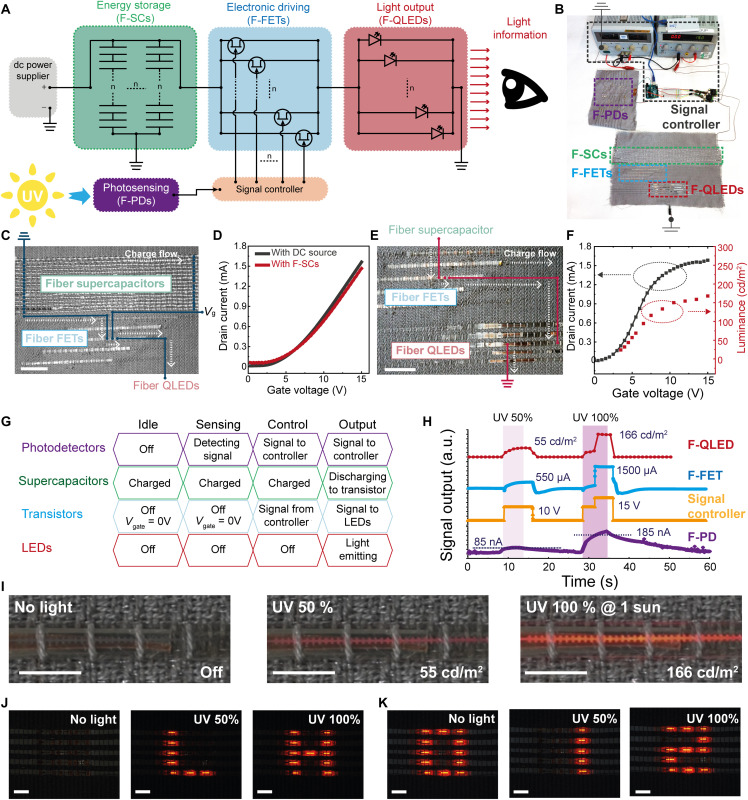
Textile electronic system for light modulation and letter indication corresponding to the intensity of incident sunlight. (**A**) Schematic of the textile system with a combination of F-PDs, F-SCs, F-FETs, and F-QLEDs, for generating environmental information by light modulation. (**B**) Photograph of the textile electronic system designed to modulate light emission corresponding to the intensity of incident sunlight. Scale bar, 10 cm. (**C**) Photograph of 16 F-SCs array and 6 F-FETs. F-SCs and F-FETs connected in series and in parallel, respectively. A signal controller is connected at the gate electrodes of F-FETs via a conductive thread. Scale bar, 2 cm. (**D**) Transfer curves of F-FETs (6 transistors in parallel) powered with either F-SCs (16 supercapacitors in series) or an external dc power supply to the drain electrode. (**E**) Photograph of the F-FETs and F-QLEDs connections. Scale bar, 2 cm. (**F**) Brightness profile of F-QLED pixels driven by *I*_DS_ of F-FETs as a function of *V*_GS_. The anode of the F-QLED is connected to the source of the F-FET, and a voltage of 16 V is applied to the drain electrode of the F-FET. (**G**) Operation chart of light modulation in four phases: idle, sensing, control, and output (light). (**H**) Output signals in the time domain of F-PD, signal controller, F-FET, and F-QLED, corresponding to the intensity of UV light arising from a solar simulator (14.7% UV in the generated light) and a 50% UV filter. (**I**) Photographs of the textile system showing the change of F-QLED brightness corresponding to the incident UV light (0, 50, and 100%). Scale bars, 1 cm. Photographs of the textile electronic system with pixelated F-QLEDs (5 × 3) displaying the information of (**J**) alphabetical and (**K**) numeric character corresponding to the incident UV light (0, 50, and 100%), Scale bars, 1 cm.

An example of an integrated textile electronic system with an architecture tailored for the AAL environments is designed with four functional fiber device blocks (F-PDs as an input device, F-SCs as an energy storage, F-FETs as an electrical driving device, and F-QLEDs as an output device) and is given in Fig. 5A and fig. S21. This system is to show the capability of the light modulation of F-QLEDs with respect to the intensity of incident sunlight onto the F-PD by an electrical driving circuit of F-FET and F-SC. Figure 5B displays the proposed textile electronic system with F-PDs, F-SCs, F-FETs, and F-QLEDs, whereas fig. S22 shows the output current of F-PDs under different sunlight intensities.

To verify simultaneous operation between fiber devices in the system, two types of system architectures are investigated: (i) F-SCs with F-FETs to show the suitability of F-SCs as a power source for the driving circuit of F-FETs and (ii) F-FETs with F-QLEDs to modulate the luminance of F-QLEDs by gate voltage (*V*_GS_) of F-FETs. Figure 5C shows 16 F-SCs and 6 F-FETs woven into the textile. Note that the electrical connection between fiber devices is completed by conductive threads. All the F-SCs with a length of 45 cm (active length, 30 cm) are connected in series and enable the generation of an operational voltage of up to 16 V. Six F-FETs are connected in parallel to increase the output current. The drain electrodes of F-FETs connected in parallel are wired up to the anode of the F-SC array through conductive thread paths. The *V*_GS_ modulation is performed by the signal controller. Transfer characteristics of the F-FETs with power supplied from an external dc power source and from the built-in F-SCs are compared in Fig. 5D, being clear that the F-SCs can effectively power the F-FETs. Figure 5E displays the integration of F-QLEDs and F-FETs in a single textile. Each source electrode of the F-FET is wired up to the anode of the F-QLED, and the cathode of the F-QLED is connected to the ground. In Fig. 5F, the luminance of an F-QLED is modulated by adjusting *V*_GS_ applied to the F-FET. With increasing drain current (*I*_DS_), brighter luminance of the F-QLED is achieved (the maximum luminance is 168 cd/m^2^ at *V*_GS_ of 15 V). These results evidence the successful implementation of the textile system with multiple types of fiber devices, showing the light modulation at the system level through a well-defined electrical layout of driving electronics.

There are four phases that describe the status of each device in the integrated textile electronics system described above: (i) idle, (ii) sensing, (iii) control, and (iv) output (light), as shown in Fig. 5G. In the first phase, F-PDs, F-FETs, and F-LEDs are off. This is the condition without any incident sunlight on the photosensors. F-SCs, when fully charged, continuously supply 16 V to the F-FETs. In the second phase, an F-PD detects the UV radiation in the sunlight, generating an output current (fig. S22), which becomes the input signal at the controller (fig. S23) while the F-FETs and F-QLEDs remain in the off state. In the third phase, the *V*_GS_ of F-FETs is controlled by the signal controller while the current is delivered from the F-PD. In the fourth phase, the current to the F-QLED is supplied by the array of F-SCs that is connected to the drain electrodes of F-FETs. The driving current of the F-QLED is determined by the *V*_GS_ applied to the F-FETs.

To investigate the environmental response of the textile system, standardized 100% UV light is generated by a solar simulator (100 mW/cm^2^, AM 1.5G), and a calibrated 50% UV filter is used to control the intensity of UV light from the sunlight. The photocurrent from F-PDs over 85 nA (50% UV) or 185 nA (100% UV) corresponds to the input voltage of 10 or 15 V that is applied to the gate of F-FETs, respectively. When *V*_GS_ is applied to the F-FETs, driving currents of 550 μA (at 10 V) and 1500 μA (at 15 V) are supplied from the F-SCs to an F-QLED through F-FETs. The luminance of the F-QLED is 55 and 166 cd/m^2^, respectively (Fig. 5H). Figure 5I shows photographs of the textile electronics system exhibiting controlled red emissions from the F-QLED, corresponding to no sunlight and 50 and 100% UV conditions. Light modulation of green F-QLED with respect to the intensity of incident sunlight is presented in fig. S24, and operation of the integrated textile electronic system with red and green F-QLED is shown in movie S4.

In addition to the textile system described above, another prototype exhibiting different characters composed of F-QLEDs in response to different sunlight intensities is also realized (fig. S25 and movie S5). The system is designed with two functional fiber device blocks, F-PDs and pixelated F-QLEDs (15 pixels; 5 × 3), controlled by an external signal processor. When 50 or 100% UV conditions of sunlight are incident on the F-PDs, the pixelated F-QLEDs show the characters of “L”/“1” or “H”/“2”, respectively. Photographs in Fig. 5 (J and K) show the textile electronic system indicating informative characters, corresponding to different incident light conditions (no light, 50% UV, and 100% UV).

## DISCUSSION

The integrated textile electronic system proposed here is demonstrated via integration on a smart textile manufacturing platform, achieving specific and scalable functionalities such as photosensing, electrical driving, energy storage, and light emission. The approach is demonstrated via automated methodologies for integration of fiber devices into the textile systems exhibiting multiple functions with the successive operations that can be adapted to a wide range of optoelectronic applications. This class of technology is fundamentally different from previous attempts to integrate electronics onto textiles substrates, which follow conventional planar electronic integration and therefore result in limited scale, limited function, and limited size. The use of fiber-based functional devices in this study is a paradigm shift from two-dimensional devices to one-dimensional fiber devices, enabling them to be directly integrated into a textile through highly scalable automated weaving and interconnection. Besides, the developed outcomes bridge the gap of technological readiness between conventional textile manufacturing and modern electronics technology, which, furthermore, is an important step toward the convergence of textile engineering and future electronic platforms.

## MATERIALS AND METHODS

### Devices fabrication

First, an F-PD, as a photosensing component, was fabricated by a dip coating method using ZnO solution mixed with Isopropyl alcohol (IPA) with 2.5 weight % and viscosity 3 centipoise (cP; Sigma-Aldrich), as shown in fig. S1. Al interdigitated patterns were deposited on a polyethylene naphthalate (PEN) planar fiber with a width of less than 2 mm (*W*), length of larger than 100 mm (*L*), and thickness smaller than 100 μm (*T*) using a thermal evaporator with a shadow mask. The ZnO layer was coated three times with a withdrawal speed of 60 mm/min on the resulting fiber, ensuring a channel dimension of 100 μm by 15 mm.

Second, an F-SC, as an energy storage component, was fabricated as shown in fig. S3. The carbon fiber, approximately 500 μm in diameter, from easycomposites comprised a bundle of 3000 carbon filaments (~7 μm of diameter), which was used both as electrodes (anode and cathode) and as the current collector. To fabricate F-SCs with a solid state electrolyte, first, poly(vinyl alcohol) (PVA) was dissolved in the deionized water with a concentration of 150 mg/ml. The mixture was stirred at 150°C for 1 hour, followed by adding phosphoric acid (H_3_PO_4_) to the mixture with a concentration of 10% v/v. The water-based PVA-H_3_PO_4_ solution as a gel-electrolyte was stirred at 150°C for 4 hours. The gel electrolyte was brushed onto the surface of carbon bundles, followed by a drying process for 4 hours at room temperature. This procedure was repeated three times to avoid undesired short circuit between the carbon fiber electrodes when two electrodes were symmetrically twisted. Then, the gel electrolyte was brushed again onto the twisted (five turns per 2.54 cm) structure of carbon/electrolyte fibers. After a drying process at room temperature for 4 hours, the F-SC with diameter of 1 to 2 mm and 45 cm long was obtained (active length, 30 cm).

Third, an F-FET with an indium-gallium-zinc oxide (IGZO) channel was prepared by vacuum deposition and photolithography, to be used as an electrical driving component (fig. S5). The fabrication of the devices was started with lamination of PEN on a 10 cm–by–10 cm glass carrier, using an adhesive developed in-house. F-FETs have a staggered bottom-gate structure, with the following layers: 80-nm-thick electron-beam evaporated Al gate electrodes, 180-nm-thick sputtered gate insulator comprising a multilayer stack of Ta_2_O_5_ and SiO_2_, 30-nm-thick cosputtered IGZO semiconductor as a channel layer, 80-nm-thick e-beam evaporated Al source-drain electrodes, and 1-μm-thick chemical vapor deposited Parylene-C for passivation. All layers were deposited without substrate heating. The patterning of the channel and electrodes was achieved by UV photolithography. SF6-based and O_2_-based dry etching were used to etch Ta_2_O_5_-SiO_2_ and Parylene-C, respectively. Width-to-length ratio of the F-FETs was 80:20 μm. Two annealing steps were used on a hot plate at 180°C for 1 hour: the former after IGZO patterning, the latter after passivation. A planar fiber having width of less than 2 mm, length larger than 100 mm (*L*), and thickness smaller than 100 μm (*T*), with seven F-FETs being then cut by laser scriber, followed by mechanical delamination from the glass carrier.

Fourth, an F-QLED as a lighting component was fabricated by a dip coating method, as shown in figs. S6 to S9. The F-QLEDs were fabricated on a polyethylene terephthalate (PET) planar fiber with a width of less than 2 mm (*W*), length larger than 100 mm (*L*), and thickness smaller than 100 μm (*T*), where a multilayered device consists of an anode, hole injection layer (HIL), hole transport layer (HTL), emissive layer, electron transport layer (ETL), and cathode. Two types of poly(3,4-ethylenedioxythiophene)–poly(styrenesulfonate) (PEDOT:PSS) were used for the electrode (anode) and HIL. The anode was prepared by highly conductive PEDOT:PSS (PH1000, Clevios) mixed with dimethyl sulfoxide to a concentration of 40% v/v with Zonyl surfactant (FSO-100, 0.01% v/v). Before the coating, O_2_ plasma was performed for 10 min to clean the surface and improve the wettability of PEDOT:PSS on PET. The anode was coated with a withdrawal speed of 2 mm/min on a PET fiber using the prepared solution. After the coating, annealing in a convection oven at 150°C for 30 min was conducted. These processes were repeated with three cycles to achieve low sheet resistance (approximately 250 ohms/sq). PEDOT:PSS solution (Al4083, Clevios) added with IPA with a concentration of 10% v/v was prepared for HIL deposition. The anode-coated PET fiber was dipped into the solution and then lifted up with a withdrawal speed of 2 mm/min. A resulting film was annealed in the convection oven at 150°C for 30 min. Poly({9,9-dioctylfluorenyl-2,7-diyl}-*co*-{4,4-[*N*-(4-*sec*-butylphenyl)] diphenylamine}) was dissolved in chlorobenzene with a concentration of 8 mg/ml as an HTL and coated with a withdrawal speed of 25 mm/min on the PEDOT:PSS–coated fiber. The sample was annealed in the vacuum oven at 150°C for 30 min. The powders of Cd-based emissive QDs were purchased from Sigma-Aldrich, and the solution of QDs was prepared in hexane with a concentration of 12.5 mg/ml. The QD emissive layer was coated with a withdrawal speed of 60 mm/min on the HTL layer and then annealed in the vacuum oven at 80°C for 10 min. ZnO solution, as an ETL material, in ethanol with a concentration of 25 mg/ml was prepared by a method reported previously (*30*). The ZnO layer was deposited with a withdrawal speed of 50 mm/min. Last, aluminum (Al), as a cathode, was deposited on the aforementioned stacking structure on the PET fiber using a thermal evaporator. The shape of fiber platform for each device was selected considering their functionalities and mechanical freedom in the textile.

### Device encapsulation

During the automated weaving process, fiber devices suffer from mechanical damages caused by the impact of the reed during battening. Encapsulation is carried out to protect fiber devices, as described in detail below (fig. S10). As-fabricated F-QLEDs were encapsulated using an additional PET fiber strip. Copper pads were partially wrapped around the PET fiber strip to make extended contact and facilitate the connection between the cathodes of F-QLEDs and the conductive threads. Cellulose with adhesive covered the whole area of the devices to hold the physical connection of the devices and Cu pad on PET. As-fabricated F-FETs were passivated by 1-μm-thick Parylene-C, as described in the device fabrication section above. As-fabricated F-SCs were encapsulated through a braiding technique, which was a process of interlacing three or more threads diagonally to a common axis. The braided outer shell protected F-SCagainst mechanical and chemical damage. Sixteen bobbins (each bobbin with polyester yarns with 330 dtex) were used for the braiding process. In particular, F-SCs have good mechanical properties after weaving, resulting from the inherent mechanical strength of carbon fibers (*31*) and a robust braiding structure. As-fabricated F-PDs were encapsulated using an additional PET fiber strip, which was similar to the encapsulation of the F-QLEDs. A Cu pad wrapping PET strip was attached to the F-PD by glue.

### Automated weaving process

Woven fabric is a structure consisting of two sets of yarn crossing at an angle of 90°. The yarn of every set generally runs parallel to the adjacent set. One set of yarn runs lengthwise and is called the warp, whereas the other set runs crosswise and is known as the weft. These two sets of yarn are held together by means of the interlacing force when yarns from the warp are placed above or under those of the weft, thus creating an endless number of pattern possibilities.

All weaving processes for integrating the functional components into the textile were carried out with an automated loom (CCi Tech) to demonstrate textile electronic systems in this study. The weaving process had five basic steps, as shown in fig. S12. Each step was carried out in sequence and was repeated until the predesigned dimension of the textile was completed. The loom is composed of a warp beam, harnesses, weft insertion device (such as a shuttle), reed, and cloth beam. In the process of inserting a weft thread, moving heddles in the vertical direction opened space between threads. A shuttle came to the opposite side, which was placed under weft threads through the open space between the reed and heddle. Once the shuttle arrived at the opposite end, a thread was grabbed by the shuttle. Then, the shuttle came back to the original position. In the process of battening by pushing the reed, the inserted thread was released from the shuttle and placed tightly along with the textile by pushing the reed. Then, heddles moved to the initial position to hold the inserted thread, and the reed came back to the original position. The setup of weaving used in this study was as follows. Warp polyester yarn (2/34 Nm; approximately 100 μm of diameter) was used, and the density of warp was 14 ends/cm. The number and width of the total warp were 560 and 40 cm, respectively. Weft polyester yarn (2/34 Nm; approximately 100 μm of diameter) with a density of 28 ends/cm was used. Double-faced eight shaft herringbone twill was used. The main weaving conditions were as follows. Warp tension was set at 50 N, which means 0.089 N for each warp end. The functional fiber devices have been inserted in the weft direction, while the conductive yarns are inserted in the warp direction (i.e., perpendicular to the fiber-based components) and aligned to each other to establish an electrical connection. To ensure insulation between active fibers and mechanical compliance, active component fibers are interposed between conventional yarns that are inserted by the automated weaving process. The optimized reed speed was 30 cm/s as required to ensure successful integration of fiber devices into the textile electronic system during the weaving process when we consider that the stress wave produced by the impact processing and transmitted to the fibers at the speed of *v* = (*E*/ρ)^1/2^ (where *E* is the modulus of elasticity of fiber and ρ is the density of fibers) (*32*) can damage the electronic fiber devices.

### Contactless automated laser soldering process

Contact-based interconnection such as conventional tip soldering was not compatible with the functional textile system because these techniques lead to thermal heat spreading to undesired positions of the devices and damaging them, given their small dimensions. Therefore, two contactless interconnection methods including induction welding and laser soldering were tested to check the feasibility of interconnection for the textile system, as shown in figs. S14, S15, and S26. The induction welding technique was not suitable in this work as it was difficult to limit the welding area to avoid unwanted heat transfer (fig. S15). Laser soldering was found to be the most suitable technique. Laser soldering was a two-step process that involved dispensing the silver-based conductive adhesive and solidification of the adhesive by laser curing. The silver-based conductive adhesive, ABLESTIK 2030SC, was supplied by Henkel. For dispensing the silver adhesive, a small syringe nozzle with a diameter of 200 μm was used. First, a small amount of the adhesive was dispensed on the interconnection node (1 to 3 mm in diameter) with conductive threads and electrodes of the fiber devices. When using the automatic dispensing method, either compressed air or a mechanical push pin was used to dispense the adhesive in a reproducible manner. For automated dispensation, it was found that the viscosity (11,600 cP) of the adhesive needed to be optimized to achieve a good droplet volume. Second, the curing of the conductive adhesive was performed at 150°C for 1 s. The power of the laser used in this study was 2.5 W at a wavelength of 980 nm. The laser focus was adjusted to change the beam spot size. The spot size and the focal length were 400 μm and 10 cm, respectively. During the laser curing, a real-time camera equipped with a pyrometer checked the temperature of the conductive adhesive, as shown in fig. 15A. The laser curing system allowed the programmed focus of the laser spot to the target position automatically. A series of tests to optimize the laser curing condition were conducted, as shown in fig. 15B. The optimal duration for laser curing was found to be 1 s for the set of materials used herein. If the duration of laser curing exceeded 1.5 s, the temperature of the conductive adhesive reached 210°C, which caused undesired thermal damage on the devices. For a curing duration of less than 0.5 s, the conductive adhesive did not solidify. Therefore, all interconnections were carried out at the laser curing condition of 2.5 W for 1 s. On the basis of automated interconnection laser soldering, including the time for laser assembly movement from idle to programmed interconnection positions, it was found that 100 interconnections took less than 10 min to complete, which is practical for large-scale textile systems.

## Notes

View/request a protocol for this paper from *Bio-protocol*.
