# Supplementary material for: Truly form-factor–free industrially scalable system integration for electronic textile architectures with multifunctional fiber devices
Source: Sci Adv. 2023 Apr 21;9(16):eadf4049. doi: 10.1126/sciadv.adf4049 (PMC10121163; doi:10.1126/sciadv.adf4049)
Supplement: Supplementary file 1 — Figs. S1 to S26 Legends for movies S1 to S5 [file sciadv.adf4049_sm.pdf]

Science Advances
AAAS

Supplementary Materials for

Truly form-factor–free industrially scalable system integration for electronic textile architectures with multifunctional fiber devices

Sanghyo Lee *et al.*

Corresponding author: Luigi G. Occhipinti, lgo23@cam.ac.uk; Pedro Barquinha, pmcb@fct.unl.pt; Jong Min Kim, jmk71@cam.ac.uk

Sci. Adv. **9**, eadf4049 (2023)
DOI: 10.1126/sciadv.adf4049

The PDF file includes:

Figs. S1 to S26
Legends for movies S1 to S5

Other Supplementary Material for this manuscript includes the following:

Movies S1 to S5

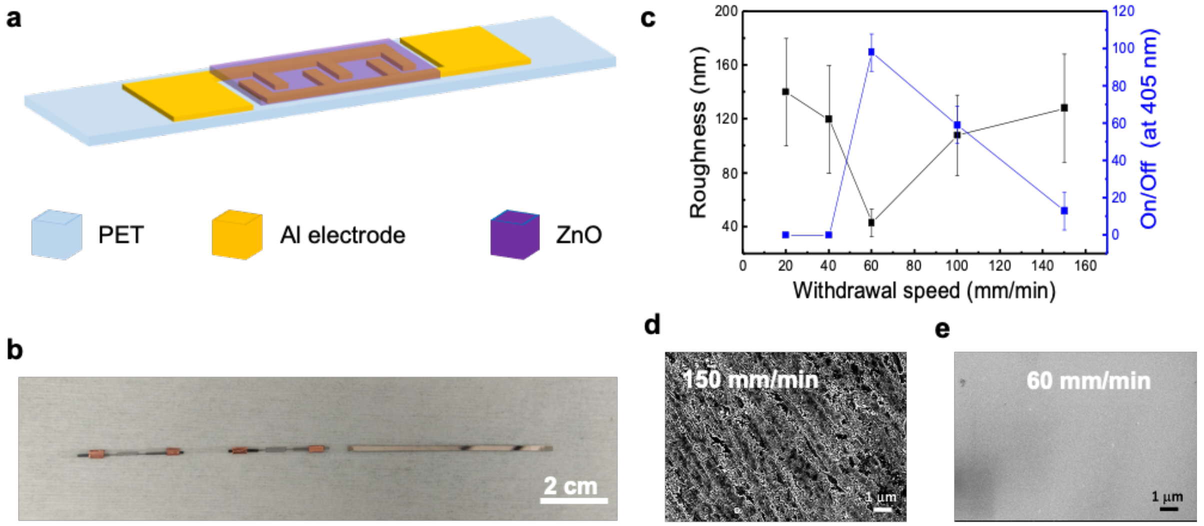

Fig. S1.

Structure and optimization of F-PDs fabricated by dip coating method. a, Schematic illustration of fiber photodetector. The active material is ZnO for UV detection. **b,** Photograph of the F-PD fabricated by dip coating method. **c,** Film roughness and the ratio of on/off at 405 nm light excitation as a function of withdrawal speed of dip coating. At a withdrawal speed of 60 mm/min, the roughness is the lowest and the on/off ratio the best among the tested conditions. Scanning electron microscopy (SEM) images showing the top view of ZnO layer fabricated with withdrawal speed of **d,** 150 nm/min and **e,** 60 mm/min.

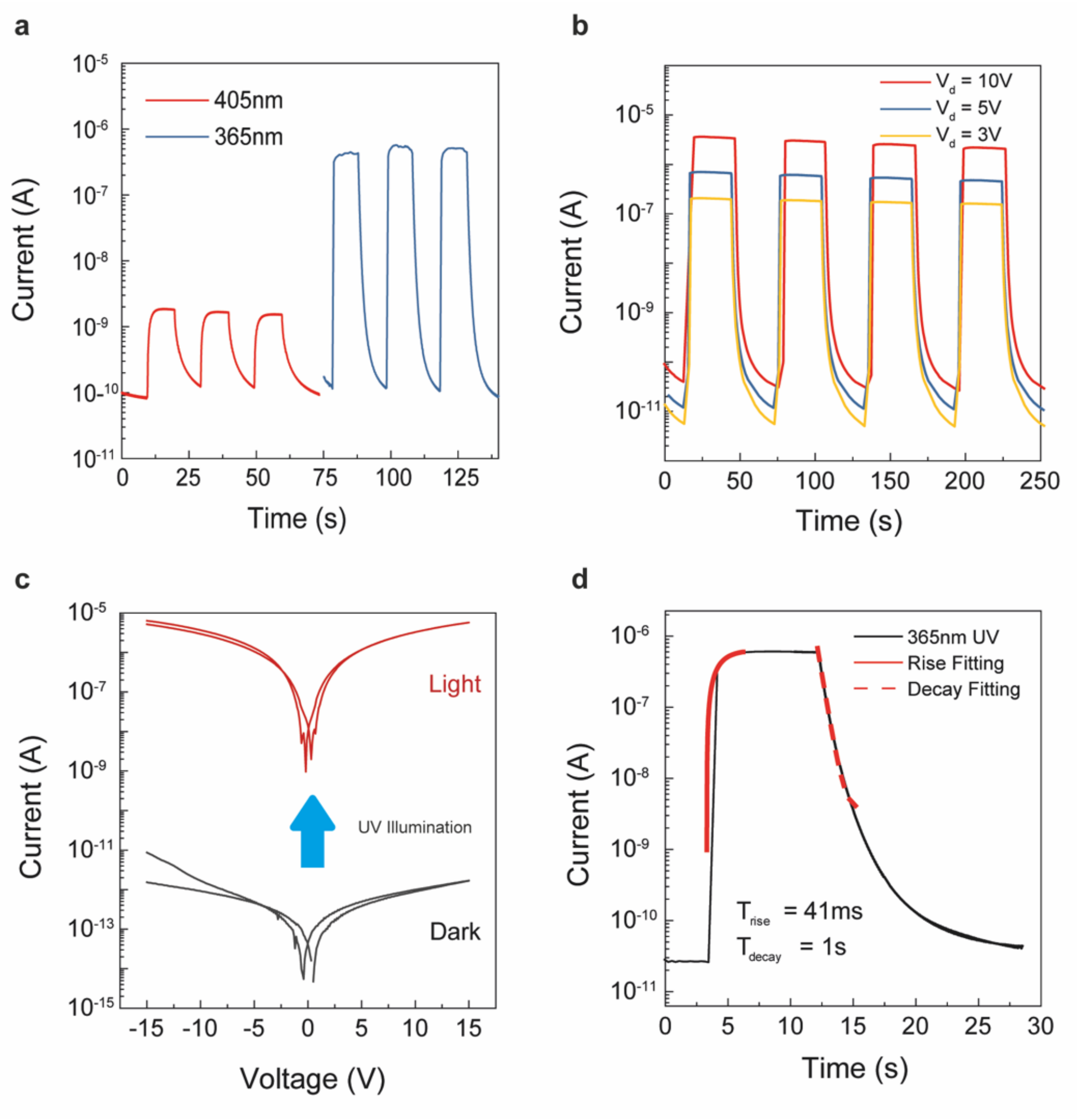

Fig. S2.

Performance of F-PDs a, Generated photocurrent of the device in time domain under excitation with light at 365 nm and 405 nm wavelength at a V_{DS} of 5 V. The on/off ratio of 10^4 is achieved. **b,** Photocurrent of F-PD with different V_{DS} in time domain under the excitation light at 365nm wavelength **c,** Photodetecting behavior of the device as a function of driving voltage with and without UV illumination at 365 nm wavelength. **d,** Response time acquired from the F-PD. The rising and decay curves in the graph are fitted using a double exponential function.

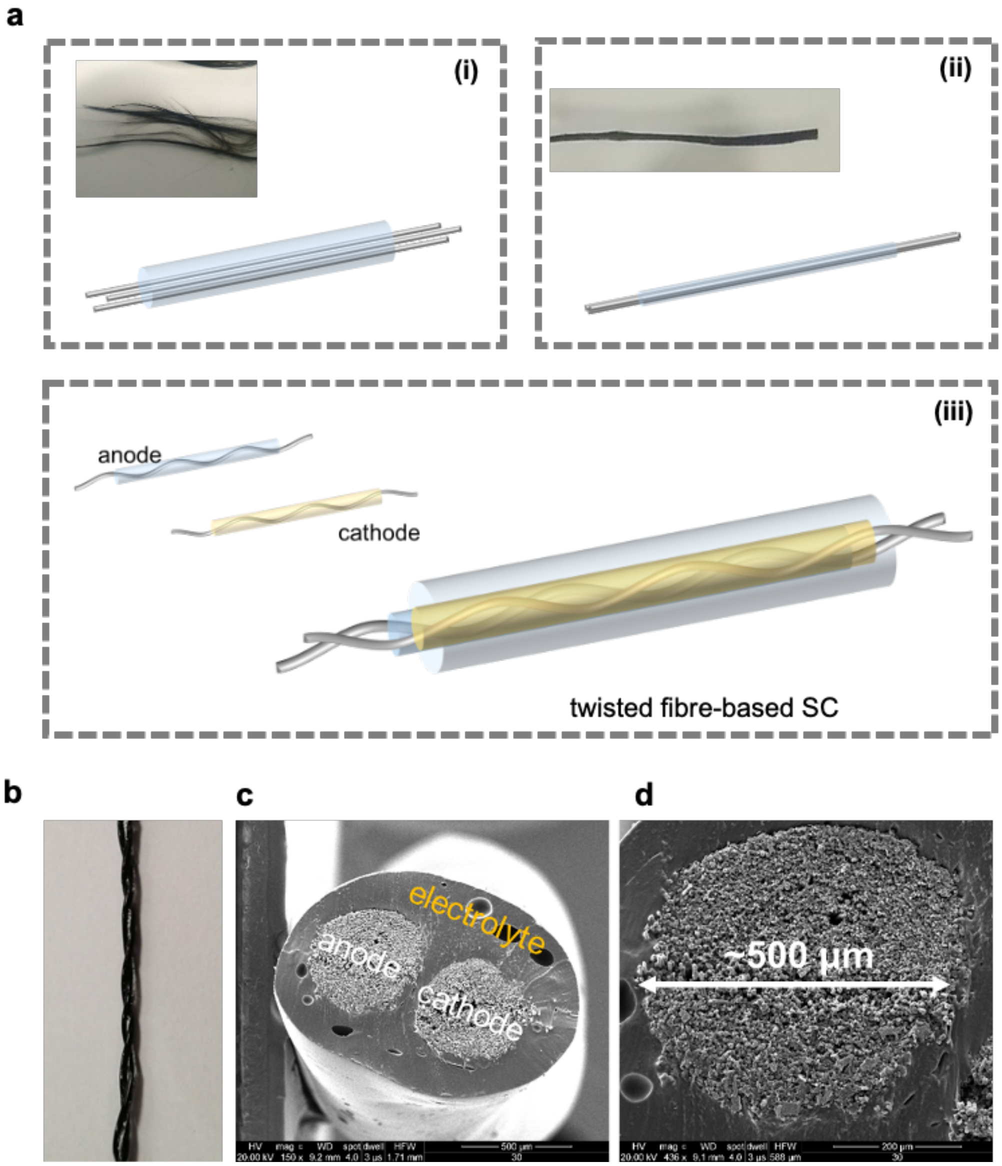

Fig. S3.

Structure of F-SC. a, Schematic illustration for the fabrication process of symmetrically twisted F-SC. (i) The bunches of carbon fibers are employed as electrodes. ii) The electrolyte is coated on the bunches and then dried. iii) The electrolyte coated bunches are twisted together and then the electrolyte is coated on the twisted structure. After drying the electrolyte, a symmetrically twisted F-SC is obtained. **b,** Photograph showing as-fabricated F-SC**. c,** SEM image displays the cross-section view of F-SC. The electrolyte is filled between anode and cathode. **d,** SEM image showing the diameter of a bunch of carbon fibers as 500 μm.

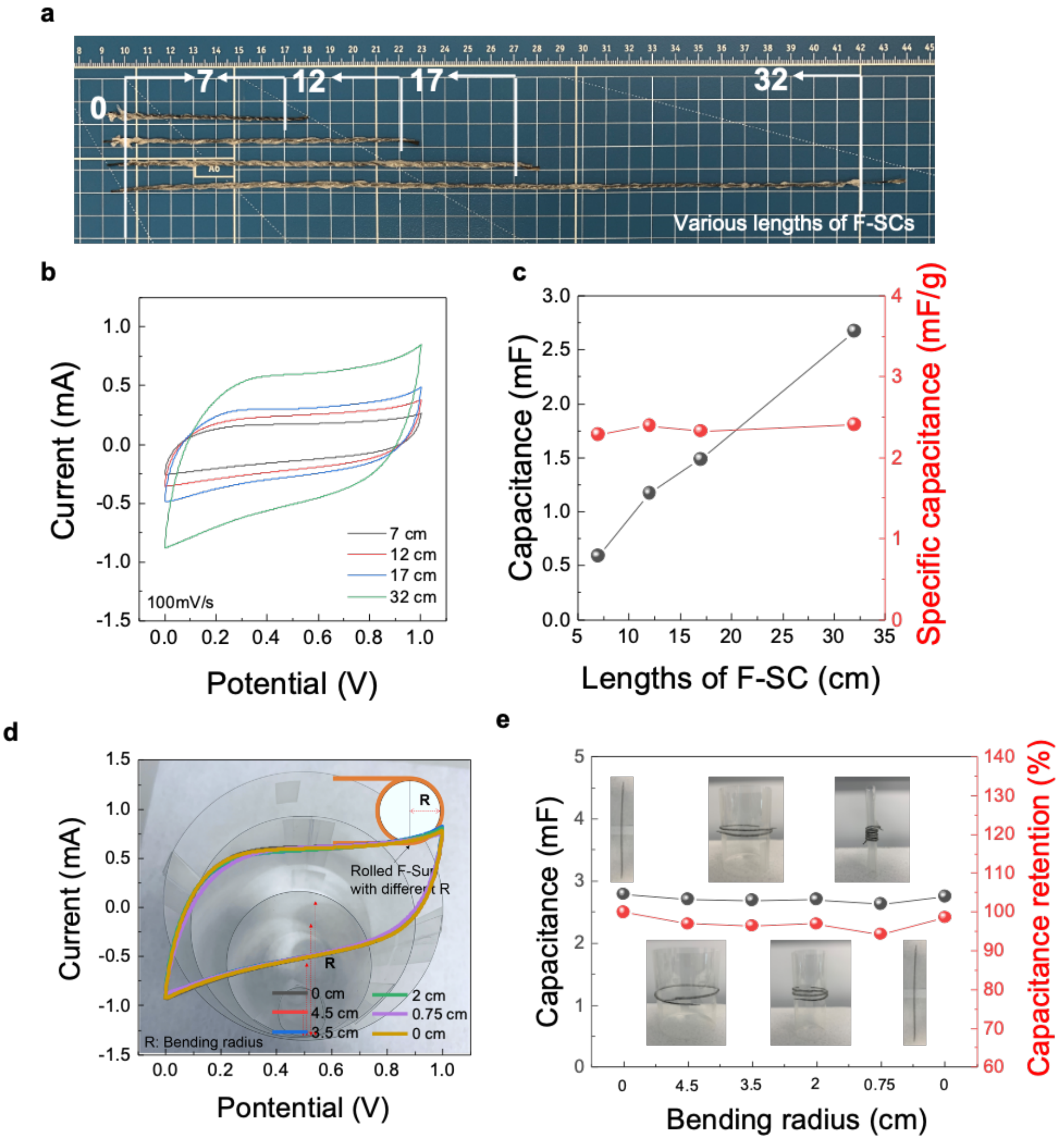

Fig. S4.

Characteristics and bending properties of F-SCs with various lengths. a, Photographs of F-SCs with various lengths, in cm. **b,** CV curves of F-SCs with different lengths at a scan rate of 100 mV/s in the potential range of 0.0 to 1.0 V. Capacitance of 2.64 mF is achieved. **c,** Capacitance and specific capacitance as a function of device length. of F-SC. **d,** CV characteristics of F-SC under various bending radii. The photographs in the background displays bending jigs. **e,** Capacitance and capacitance retention values of F-SC under various bending radii. The inset photographs show the bending states of F-SCs during the measurements.

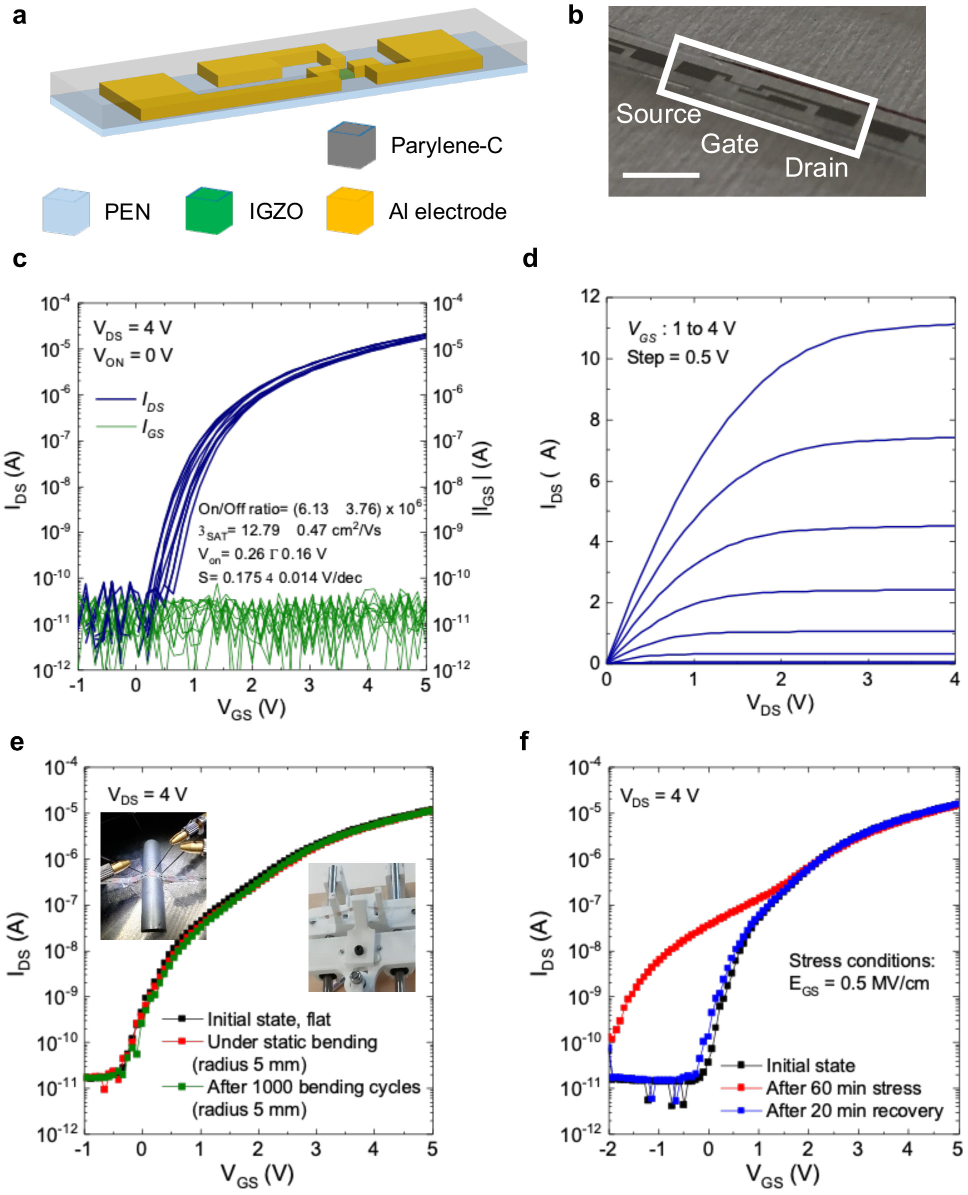

Fig. S5.

Structure and evaluation of F-FETs. a, Schematic illustration of F-FET. The devices have a staggered bottom-gate, top contact structure. The device is passivated by 1 µm of Parylene-C. **b,** Photograph showing the unit device fabricated on a PEN fiber. Each fiber is 10 cm long, 1.5 mm wide and has seven FET devices. Each FET has width, length and channel thickness of 80 µm, 20 µm, and 40 nm respectively. Scale bar, 2 mm. **c,** Transfer curves at V_{DS} = 4 V for six F-FETs, demonstrating small device-to-device variation, despite the low-temperature fabrication processes (limited to 150 °C). **d,** Output curves at different VGS in the range from 1 V to 4 V, with a step of

0.5 V. **e,** Electrical properties of the device after static bending and 1000 bending cycles, with a radius of 5 mm, revealing good mechanical reliability. The insets show the photographs of the bending setups, for static (left) and cyclic (right) measurements. **f,** Test of gate bias stress on the device with V_{GS} of 0.5 MV/cm, showing fully recoverable properties after continuous electrical stress.

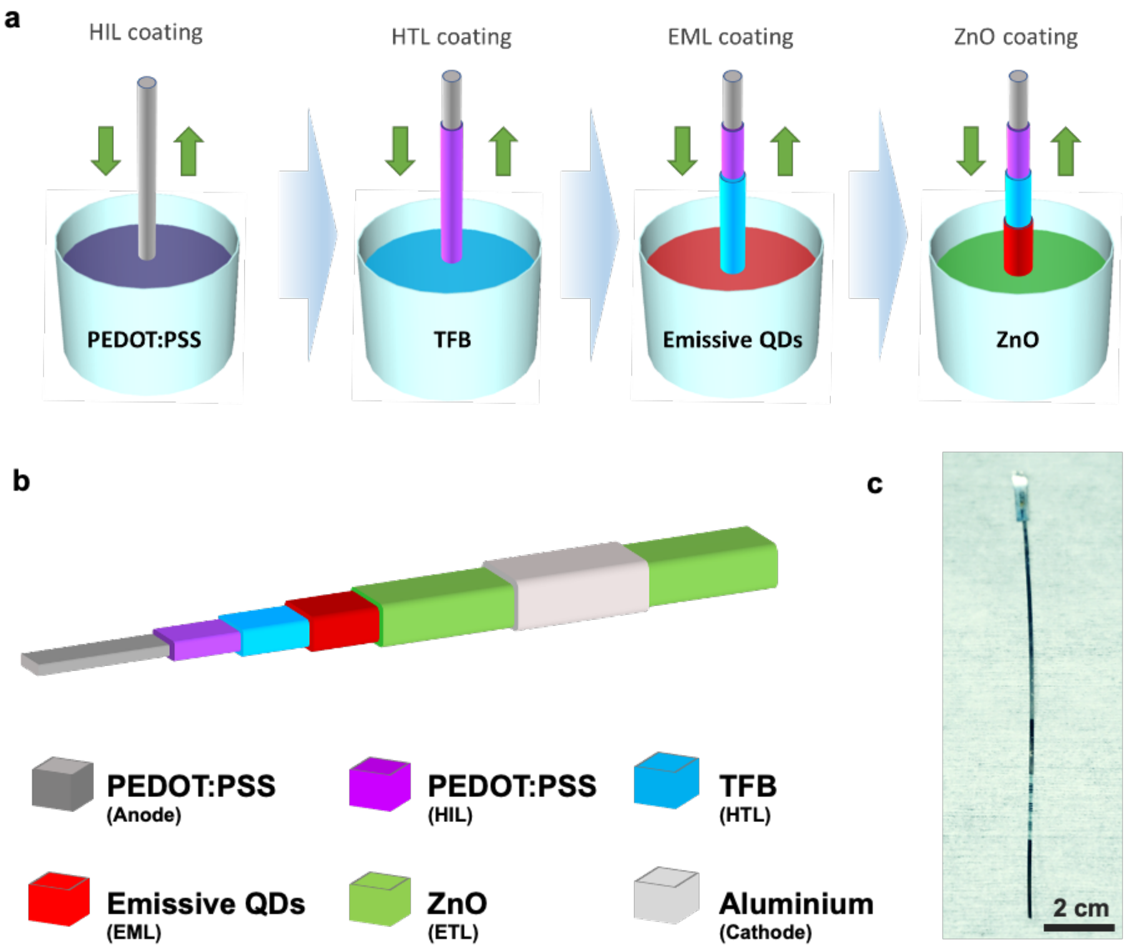

Fig. S6.

Fabrication of F-QLED. a, Schematic diagram of fabrication procedure of F-QLED by dip coating method **b,** Schematic illustration of a single F-QLED device. Six functional layers are sequentially deposited on a PET fiber substrate. **c,** Photograph of fabricated F-QLED. The width and length of the devices are 1 mm and 10 cm, respectively**.**

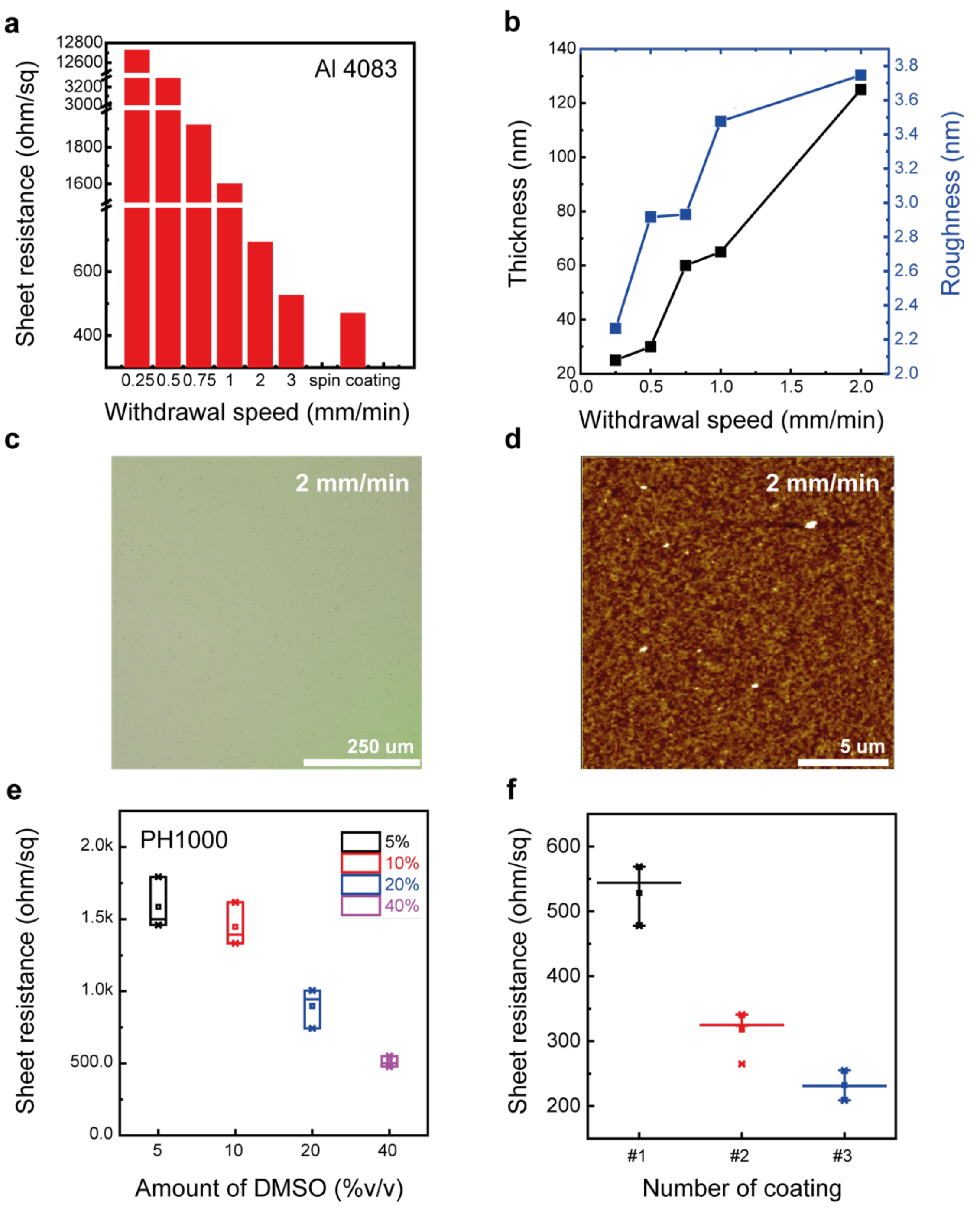

Fig. S7.

Optimization of PEDOT:PSS layer as anode and HIL by dip coating process. a, Sheet resistance of PEDOT:PSS layers (Al4083, HIL) as a function of withdrawal speed. PEDOT:PSS films with withdrawal speed of over 3 mm/min were not uniform. **b,** Thickness and surface morphology analysis of PEDOT:PSS layer (HIL) as a function of withdrawal speed. **c,** Optical

image and **d,** Atomic force microscopy (AFM) image of PEDOT:PSS (HIL) layer fabricated by dip coating method at a withdrawal speed of 2 mm/min. **e,** Sheet resistance of PEDOT:PSS layers (PH1000, anode) with the different volume ratios of DMSO at 2 mm/min withdrawal speed. **f,** Sheet resistance of PEDOT:PSS layers (PH1000 solution with 40% v/v of DMSO) as a function of the number of coatings at a withdrawal speed of 2 mm/min.

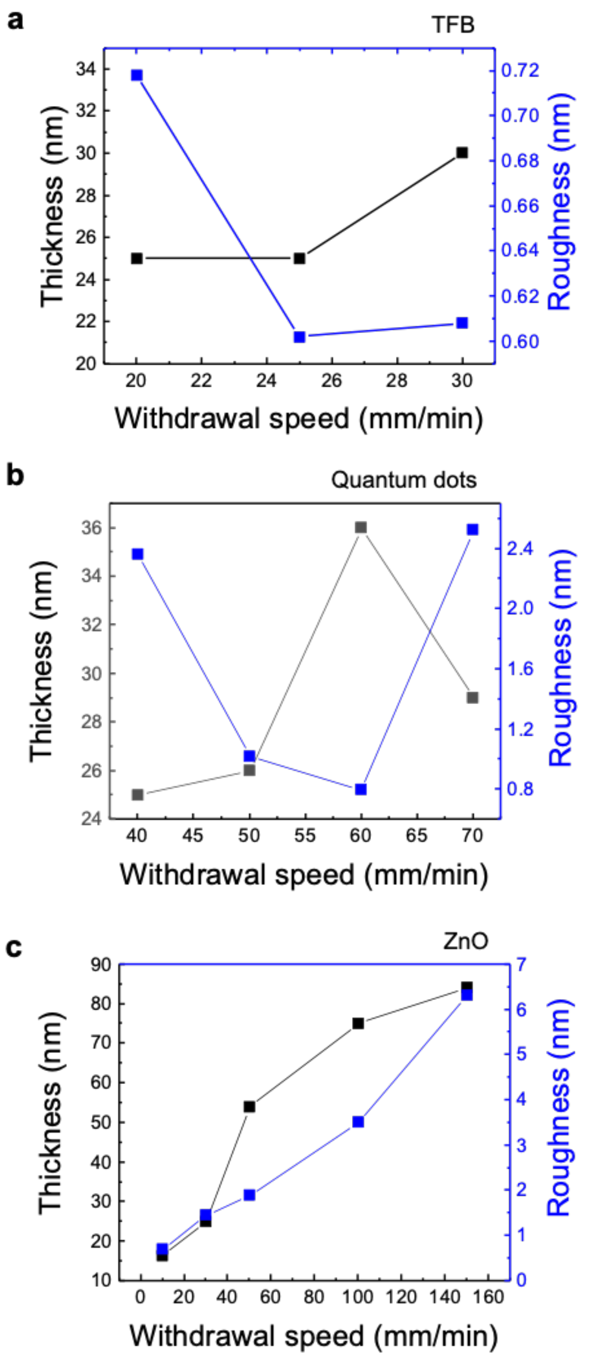

Fig. S8.

Optimization of functional layers for F-QLED. Thickness and roughness analysis of **a,** TFB layers as a HTL, **b,** QDs layers as an emissive layer, and **c,** ZnO layers as an ETL, to optimize the withdrawal speed of dip coating. All measurements were conducted by AFM.

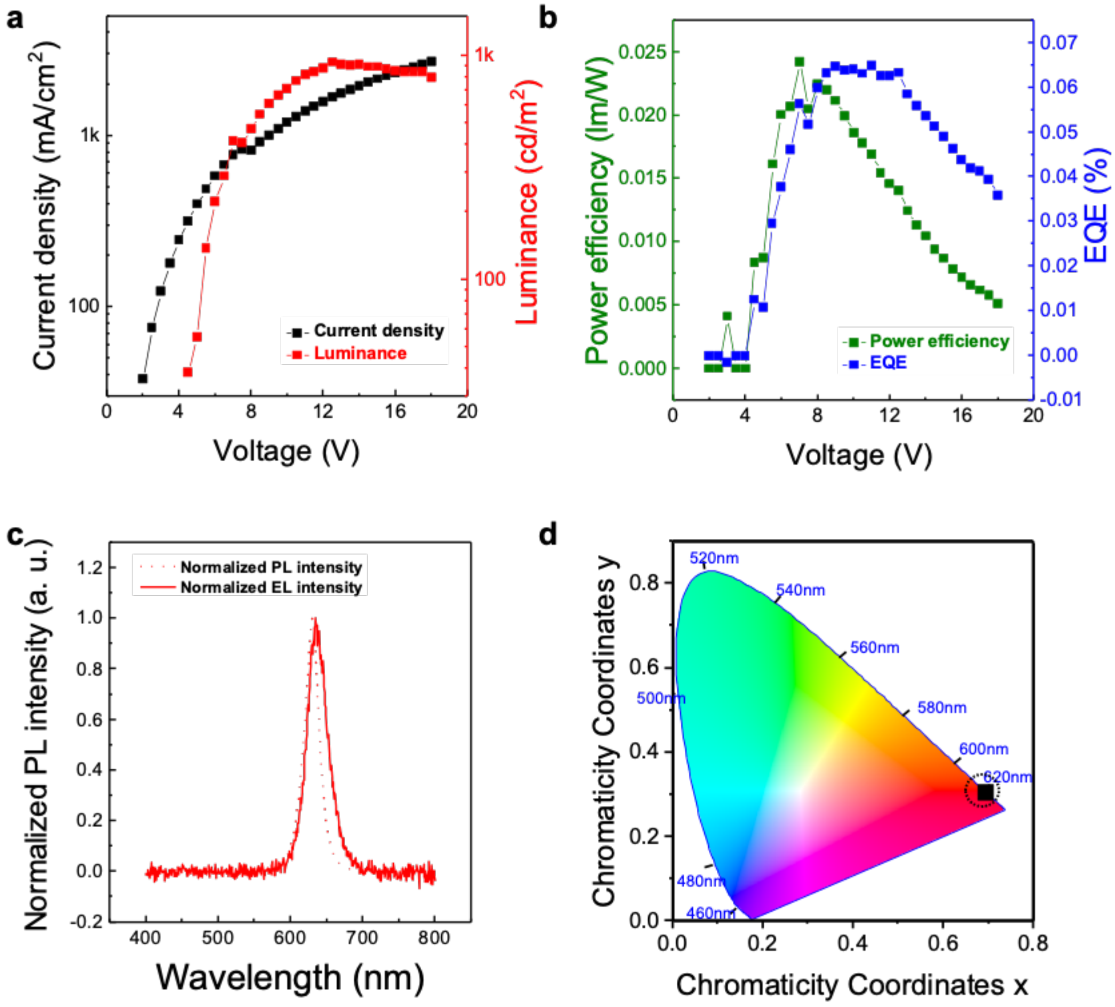

Fig. S9.

Characterization of F-QLED. a, Current density and luminance versus driving voltage. The luminance of over 900 cd/m^2 is achieved. **b,** Power efficiency and external quantum efficiency (EQE) versus driving voltage for the device. **c,** Electroluminescence spectrum for an applied voltage at 10 V and photoluminescence spectrum of the quantum dots layers. **d,** CIE 1931 coordinates for red emission of F-QLED.

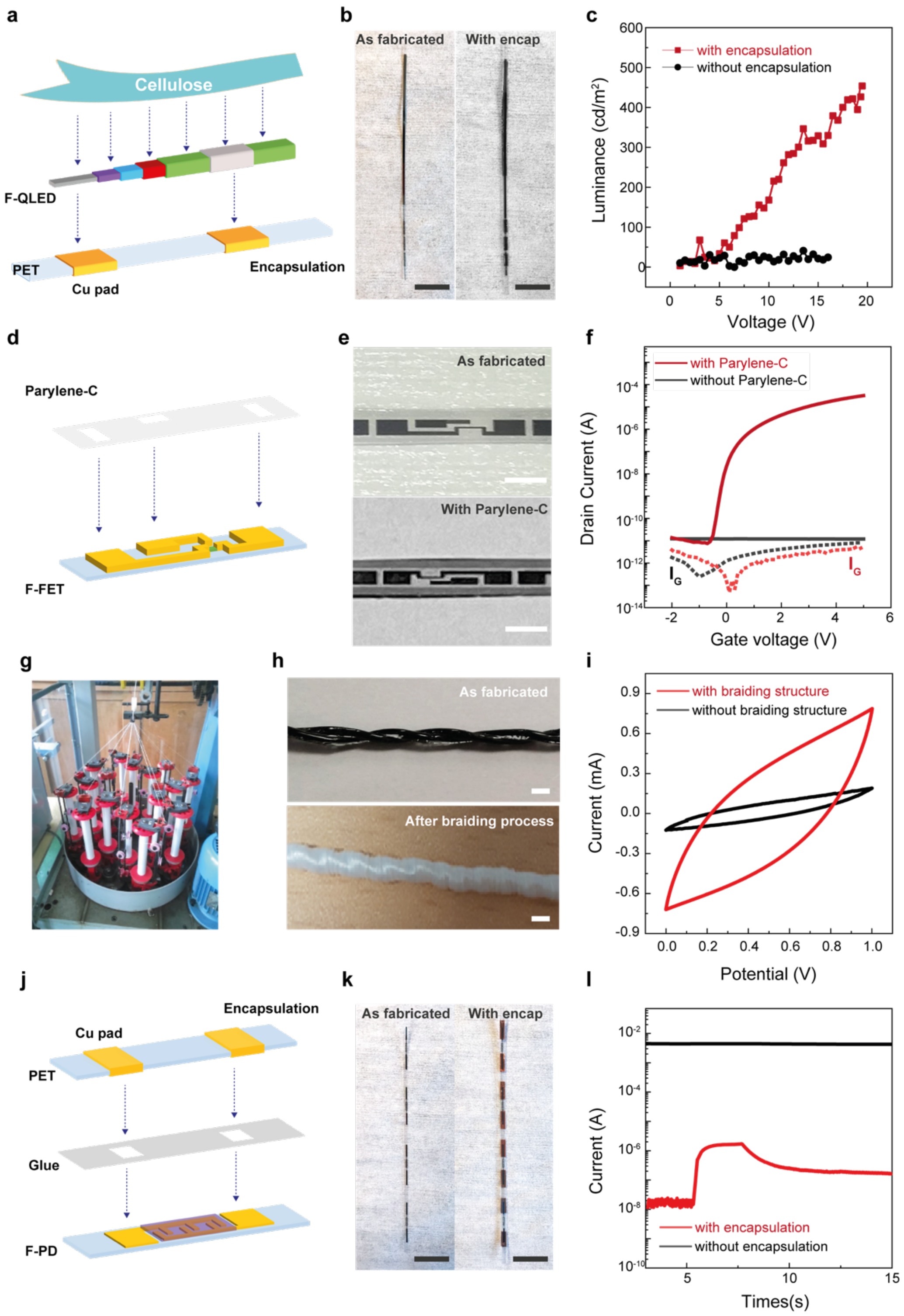

Fig. S10.

Encapsulation of the functional fiber components for the automated weaving. The characteristics of fiber devices with and without encapsulation are carried out after the weaving

process. **a,** Schematic illustration of encapsulation for the F-QLEDs. **b,** Photographs showing the encapsulated F-QLEDs after assembly with PET fiber strip and cellulose. Scale bars, 2 cm. **c,** Luminance versus driving voltages for F-QLEDs showing the failure of the device without encapsulation. The luminance of F-QLED with the encapsulation structure is 444 cd/m^2 **d,** Schematic illustration of Parylene-C passivation for the F-FETs. **e,** Photographs of F-FETs before and after passivation by Parylene-C. Scale bars, 2 mm. **f,** Transfer curves of F-FETs integrated into the textile at a V_{DS} of 4 V. Devices without passivation scheme fail to withstand the weaving process. **g,** Photograph of the equipment for the braiding process. **h,** Photographs of F-SC as fabricated and after braiding process as an encapsulation. Scale bars, 2 mm. **i,** Cyclic voltammetry (CV) curves of F-SCs at a scan rate of 100 mV/s in the potential range from 0 to 1 V. F-SC without braiding structure is damaged, revealing a decreased capacitance of 0.69 mF compared to the braided F-SC which has a capacitance of 2.84 mF. **j,** Schematic illustration of F-PD encapsulation, following a similar approach as used for F-QLED. **k,** Photographs of F-PD before and after the encapsulation. **l,** Photocurrents in the time domain acquired from F-PDs shows that F-PDs without encapsulation are damaged during the weaving process. The on/off ratio of F-PD with encapsulation is over 10^2 after the weaving process. Scale bars, 2 cm.

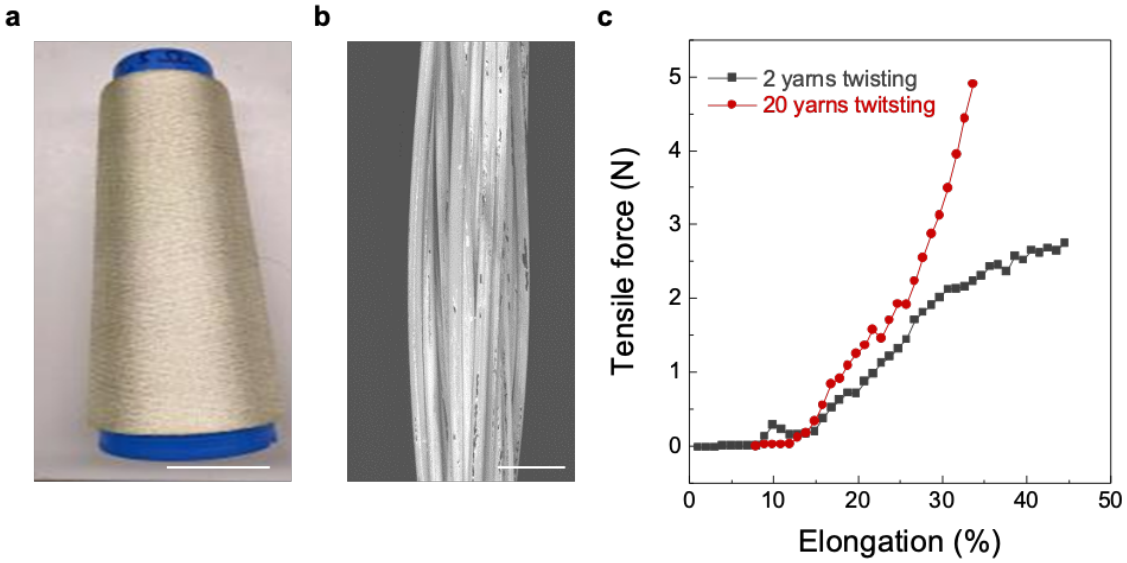

Fig. S11.

Conductive thread. a, Photograph of a bobbin of conductive thread. Scale bar, 5 cm. **b,** SEM image of surface on the conductive thread. Scale bar, 100 μm. **c,** Mechanical properties of conductive thread with regard to numbers of yarns twisting. The silver-coated polyamide conductive thread was fabricated as follow: (i) pre-treatment for seeding, (ii) electroplating, (iii) washing, (iv) drying.

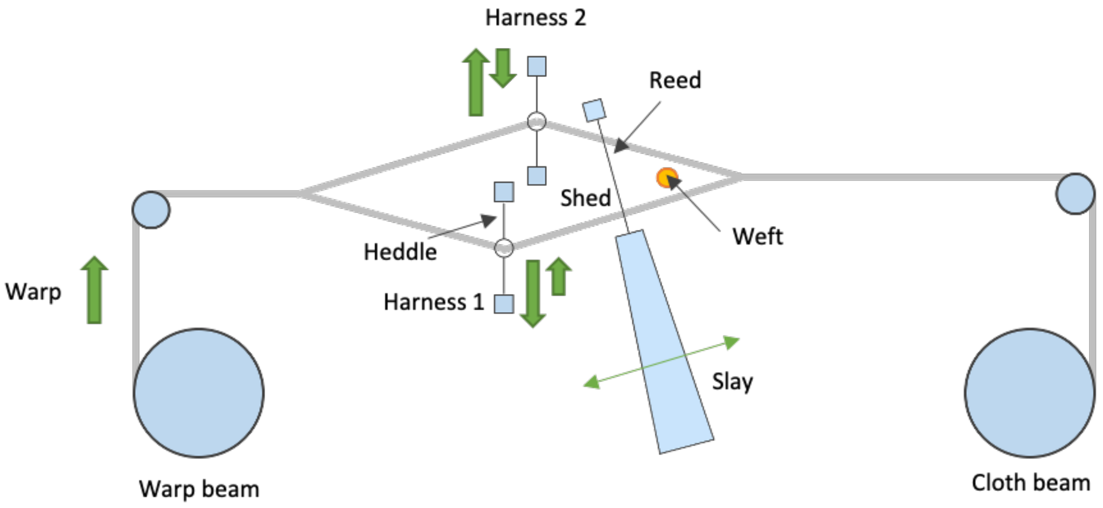

Fig. S12.

Schematic of weaving process by a loom. Schematic illustration showing the procedure of weaving. The sequence of 1 cycle of the weaving process is as follows: (i) Warp Let-off motion; the delivery of warp yarn at required speed from the warp beam which controls the tension of warp. (ii) Shedding; the process of separating warp yarn into two layers by raising harnesses 1 and 2 with the heddle to form an open area between the two layers of warps. This determines the weave pattern. (iii) Picking; inserting the weft yarn through the shed with a shuttle. (iv) Battening; the process of pushing the newly inserted weft yarn to the already woven fabric at the point of fell of the cloth with the reed. (v) Cloth Take-up motion; The winding of woven cloth at a required speed from the cloth beam which controls the tension. This sequence of steps is repeated continuously to generate the woven textile.

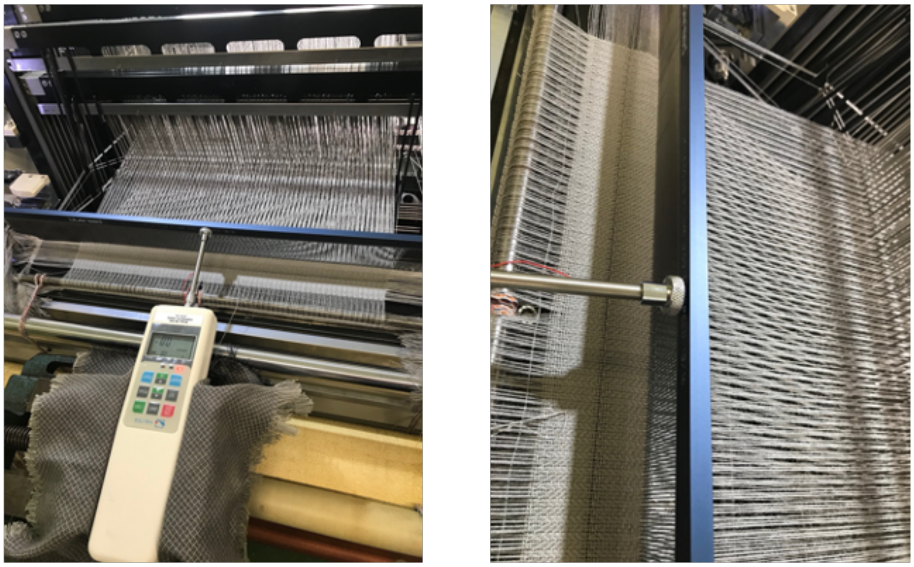

Fig. S13.

Set-up for measurement of force during weaving process. Photographs show the installation of a force meter on the weaving machine. The force meter is located at the last weft thread on which the reed impact applies.

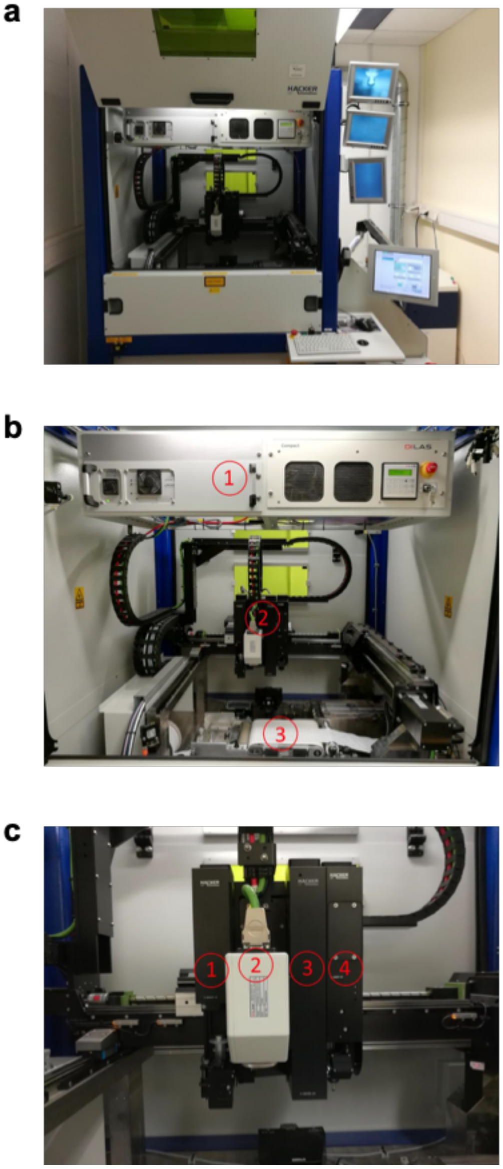

Fig. S14.

Equipment for laser welding process. a, Photo image of laser welding machine. **b,** Partition of the assembly machine (1) laser working station, (2) Gantry head, (3) working area. **c,** Gantry head modules; (1) dispense unit, (2) laser soldering unit, (3) optical camera, (4) vacuum mounting tool. This machine manufactured by Häcker Automation GmbH is generally used for placing electronic components on PCBs of all kinds and is capable of dispensing solder or other high-viscosity substances, optical recognition, laser soldering as well as picking and placing electronic components. Based on the functionality of this machine, we utilized it for the interconnection process of our textile electronics. The gantry head is the main tool for processing textiles. The

gantry head is a two-axis moving platform, which can be exactly positioned up to a micrometre inside the working area. Different modules are installed on the gantry head, which perform different functions. The gantry head is able to carry up to 5 modules for example. Those modules add a third moving axis, to adjust the height between the textiles and the modules itself. This set-up enables a precise 3-dimension system to conduct automatic processes with variable modules. Currently, the gantry head has a programmable solder dispense unit, a laser soldering unit, an optical camera, and a vacuum mounting tool.

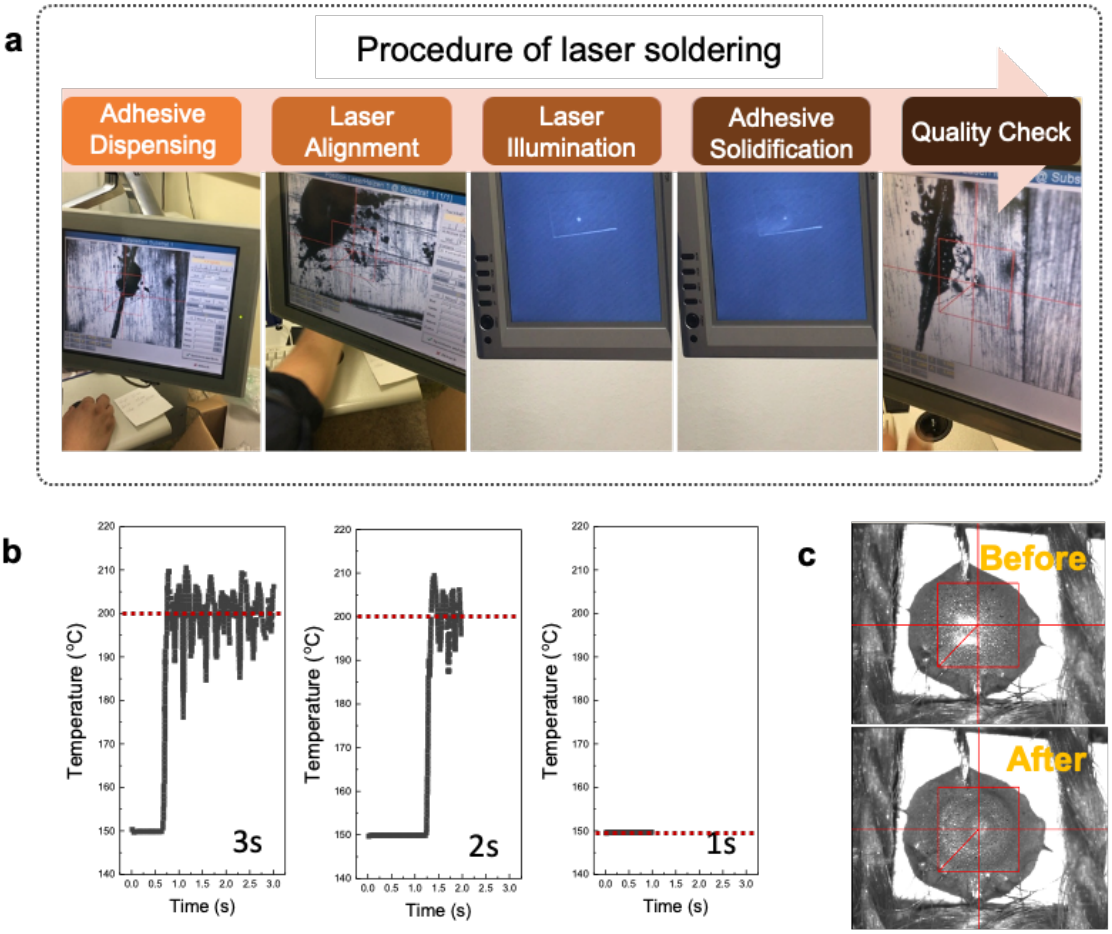

Fig. S15.

Laser welding process. a, Flow chart for the whole procedure of laser soldering. At first, the conductive adhesive is dispensed onto the target position programmed in the automation system. Then the laser is aligned at the same position automatically. The laser illuminates the adhesive to solidify it for a few seconds. **b,** Temperature profiles of the adhesive during the laser illumination with different curing times. **c,** Optical images of adhesive before and after laser curing. The IR laser heats up the silver adhesive directly.

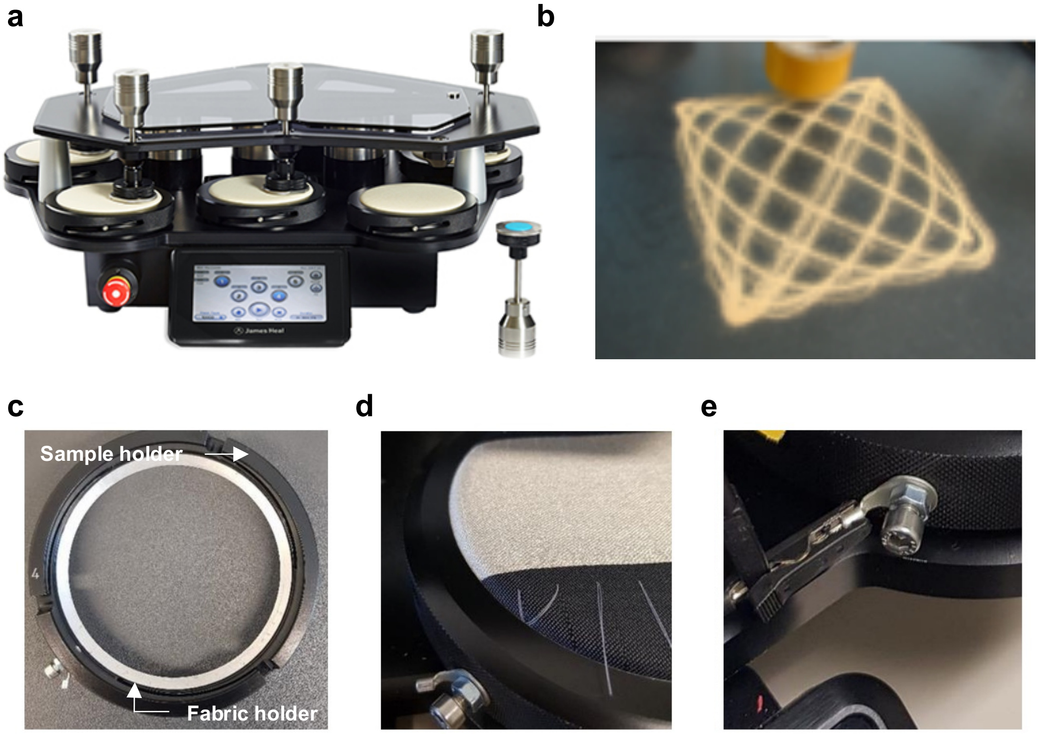

Fig. S16.

Abrasion test set-up. a, Martindale apparatus for abrasion test. **b,** Lissajous pattern for 1 cycle. **c,** Modified Martindale ring with a removed insulation layer. **d,** Mounted ring in test device with clamped dummy textile. **e,** Connection of the ring to electrical measurement set-up. The test set-up is modified to carry out 4-wire measurement of the electrical resistance for validating the interconnection, based on the DIN EN ISO 12947-2:2016. Abrasion tests are conducted continuously with a frequency of 1 Hz and the resistance is monitored simultaneously when the test is running.

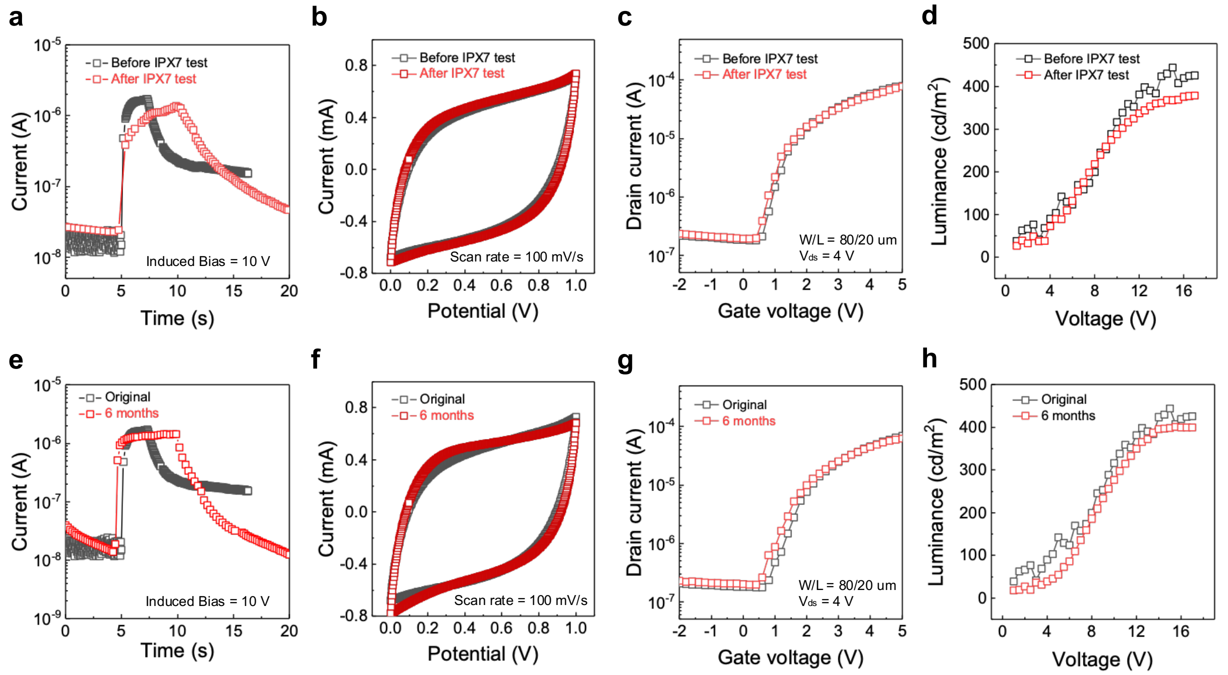

Fig. S17.

Reliability tests of fiber devices integrated into the textile through automated weaving and laser interconnection. Water resistance tests under IPX7 conditions (1 meter deep, 30 minutes) for **a,** F-PD, **b,** F-SC, **c,** F-FET, and **d,** F-QLED. Long-term stability tests after 6 months of storage in the air for **e,** F-PD, **f,** F-SC, **g,** F-FET, and **h,** F-QLED.

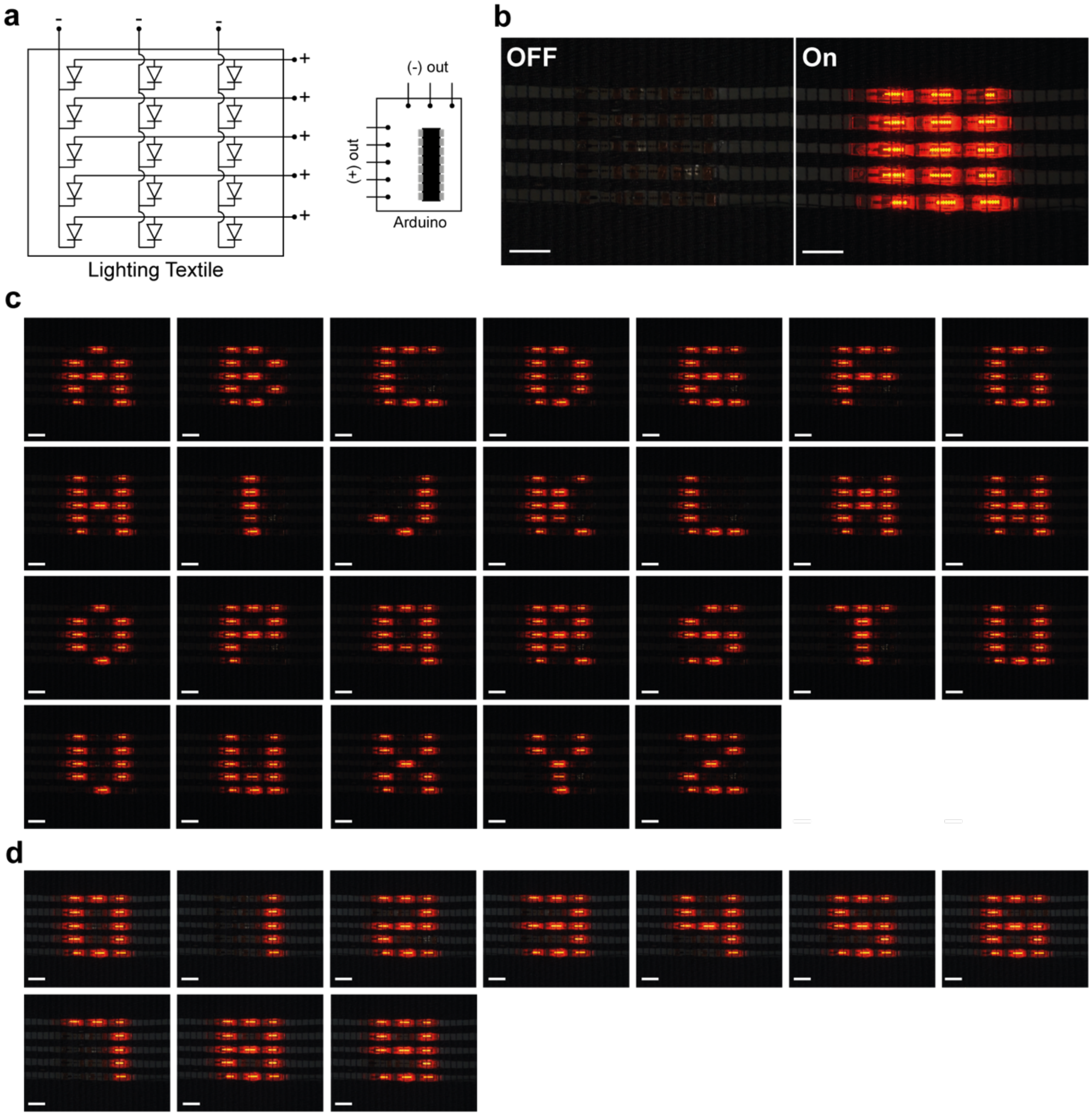

Fig. S18.

Demonstration of alphabetical characters and numbers indication with pixelated F-QLEDs woven in the textile. a, Circuit diagram of the demonstration of letter indication with pixelated F-QLEDs woven in the textile through an automated integration method. 5 × 3 pixels are prepared in the lighting textile. Arduino controls each pixel. **b,** Photographs showing entire pixels of F-QLEDs woven in the textile turned on/off. The operation voltage is 10 V. Scale bars, 1 cm. Demonstration of characters indication for **c,** capital letters of alphabet and **d,** numbers. Scale bars, 1 cm.

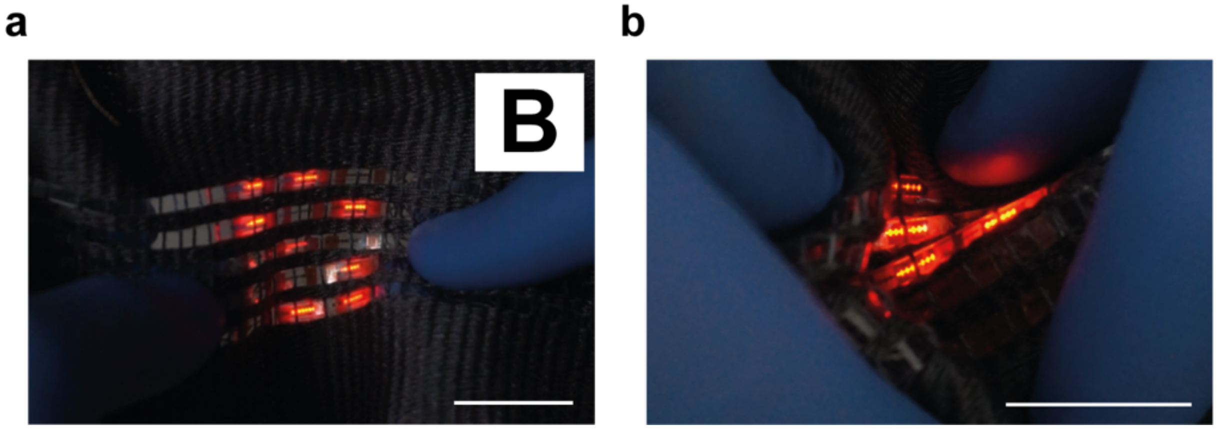

Fig. S19.

Bendable lighting textile. Photographs of **a,** lighting textile indicating B that represents the alphabetical character of "Bendable" and **b,** crumpled lighting textile. Scale bars, 2 cm.

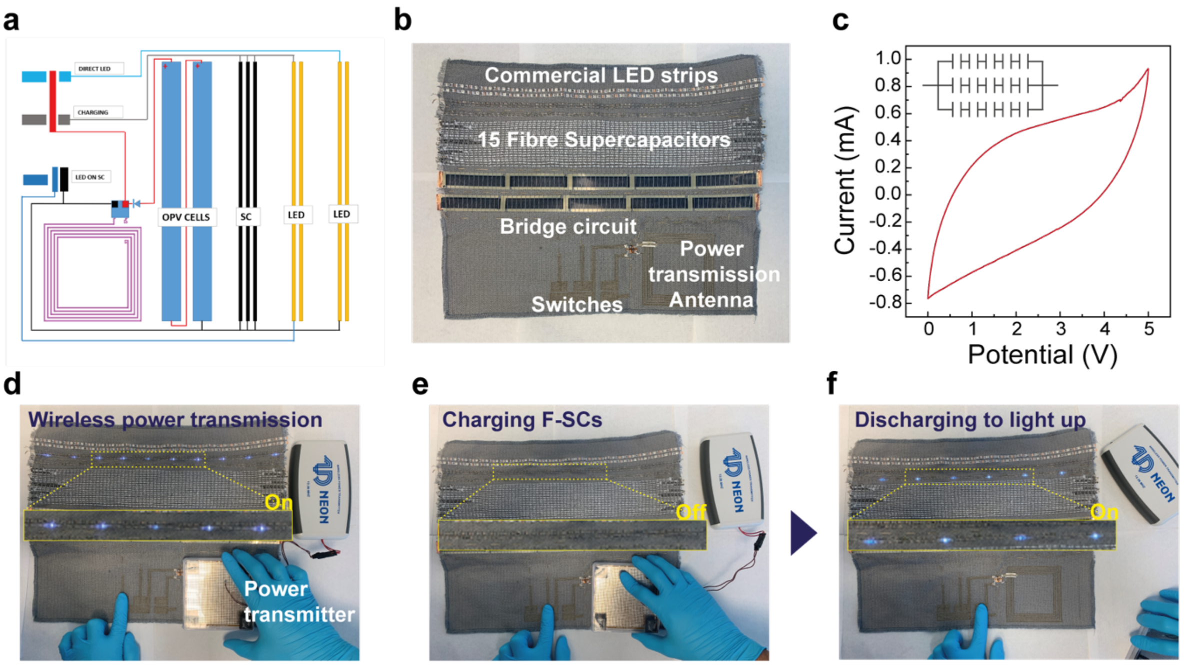

Fig. S20.

Demonstration of energy storage system charged by radio-frequency (RF). a, Schematic design of the energy management system. The demonstration comprises switches, power transmission antenna, bridge circuit, commercial LEDs, and fifteen F-SCs. **b,** Photograph showing the textile demonstration for energy management. The textile with F-SCs and LEDs is fabricated by the automated weaving method, while other electronic components such as switches and the antenna are integrated onto the textile by a programmable embroidery machine. Finally, the bridge circuit is installed on the textile and interconnections complete the circuitry. **c,** CV curve of an array of fifteen F-SCs designed and connected to generate 5 V with a capacitance of 1.70 mF at a scan rate of 100 mV/s. The inset indicates parallel/serial connections of F-SCs. **d,** Commercial LEDs powered by wireless power transmitter (2.5 mW) through a textile RF (13.56 MHz) antenna (Inductance: 2.64 μH), textile switch, and conductive threads. **e,** Photograph displaying the charging of F-SCs by wireless power transmitter through RF antenna. **f,** Commercial LEDs powered by F-SCs without any external power sources.

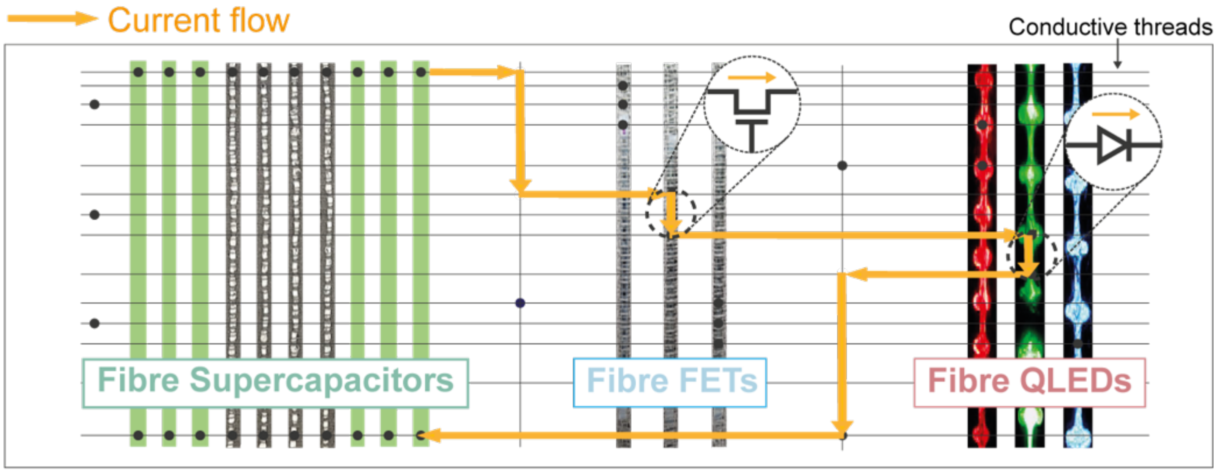

Fig. S21.

System architecture with combination of multiple fiber components. a, Schematic diagram of the textile electronic system architecture with photographs of embedded fiber devices of F-SC, F-FET, and F-QLEDs (luminance of R, G, and B= 443, 482, and 188 cd/m^2), and an electrical layout in single textile accomplished by conductive threads.

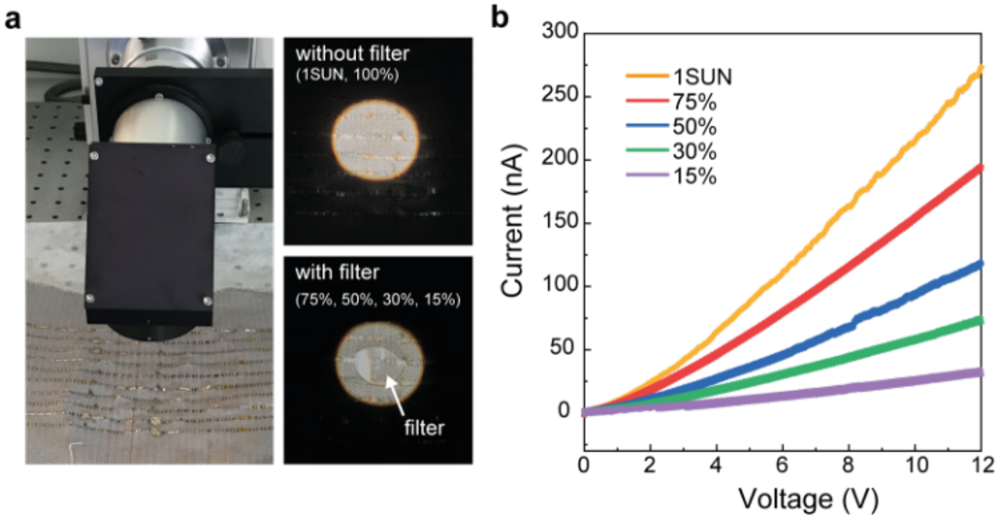

Fig. S22.

Output current of F-PDs with different intensity of sunlight. a, Photographs of measurement setup for photocurrent with different intensity of sunlight. **b,** The photocurrents are measured in a solar simulator (100 mW/cm^2, AM1.5G). UV filters allow for various levels of UV light attenuation.

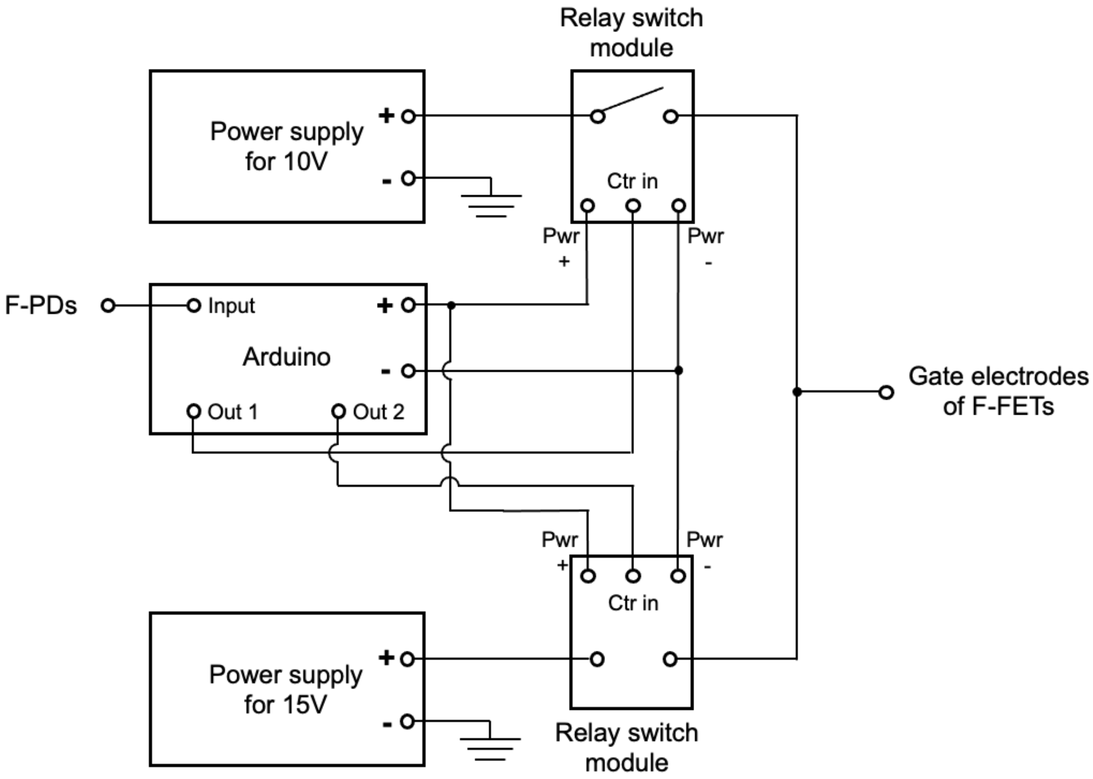

Fig. S23.

Circuit diagram of a signal controller. The signal controller consists of two power supplies, two relay switches, and an Arduino. Programmed command on Arduino operates to turn on the relay switches that are connected to power supplies, corresponding to current input from F-PDs.

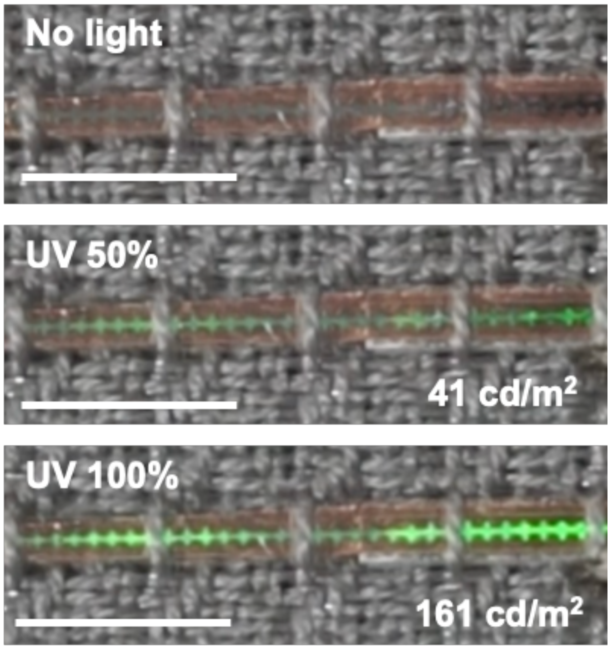

Fig. S24.

Light modulations of green F-QLED. Photographs of the textile electronic system showing the change of green F-QLED brightness corresponding to the incident UV light (0, 50, 100 %). Scale bar, 1 cm. The controller induces 3 or 5 V to the gate of F-FETs when 50 or 100% UV light was detected by F-PD, respectively.

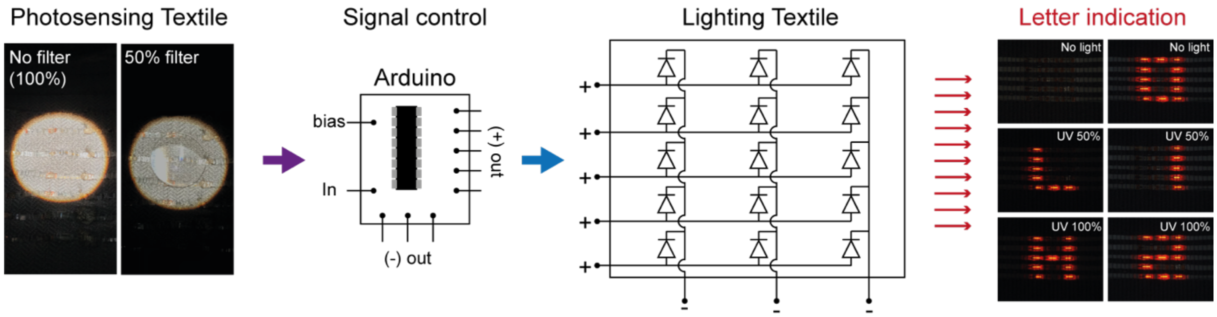

Fig. S25.

System configuration of character indication corresponding to the intensity of incident sunlight. A textile electronic system consists of F-PDs (as an input device) and F-QLEDs (as an output device), controlled by Arduino. As for the textile system in Fig. 3, standardized UV light is generated by a solar simulator and a 50% UV filter is used to attenuate the intensity of UV light in the simulated sunlight. In the Arduino controller, it is programmed that a photocurrent from F-PDs over 85 nA (50% UV) or 185 nA (100% UV) is defined as indicating “L” in the alphabetical character system / “1” in the numerical character system or “H” in the alphabetical character system / “2” in the numerical character system, respectively.

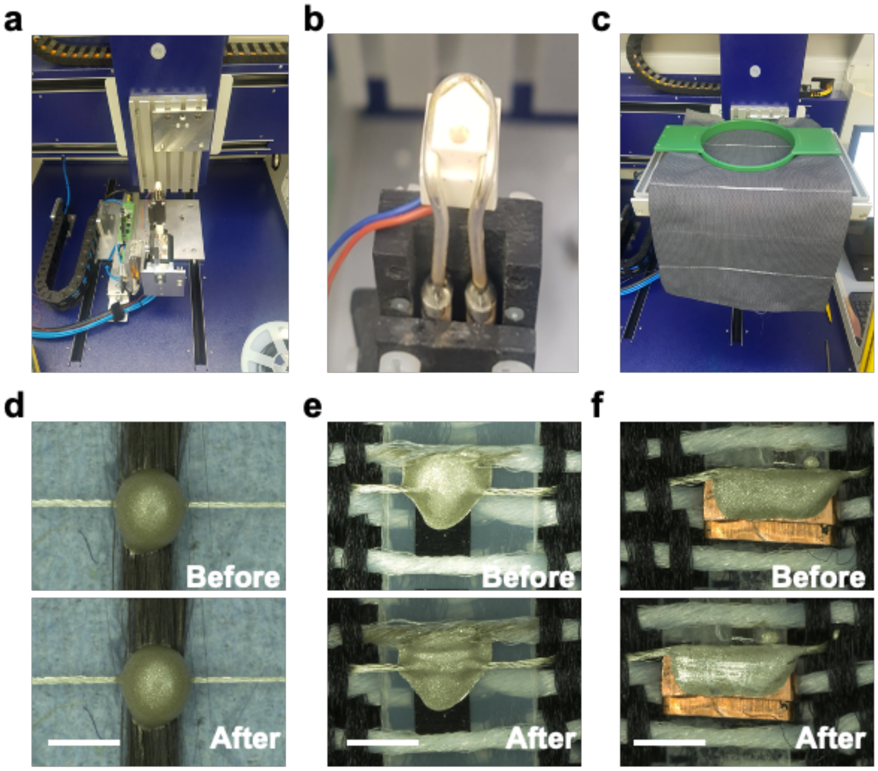

Fig. S26.

Induction welding process. Induction welding method is a contactless interconnection technique evaluated in this work. **a,** An induction welding machine equipped with a single coil and X-Y-Z movement controller. **b**, Photograph of magnified induction heater. This system allows a spatial resolution of 500 µm, defined by the specification of a step motor, and a maximum induction power of 3.5 kW. **c,** Photographs of textile loaded on the welding machine. Photographs showing silver adhesive dispensed onto the interconnection point between a conductive thread and device electrode materials such as **d**, Carbon fiber, **e**, Molybdenum, and **f**, Copper before/after the induction welding process. Scale bars, 1 mm. The process was conducted by an induction power of 700 W for 20 sec.

Movie S1. (separate file)
Weaving process for fiber components

Movie S2. (separate file)
Interconnection process by laser welding

Movie S3. (separate file)
Pixelated F-QLEDs for character indication

Movie S4. (separate file)
System operation of the textile electronic system for light modulation

Movie S5. (separate file)
System operation of the textile electronic system for character indication
